# Comparative Analysis of Phytochemical Profiles and Selected Biological Activities of Various Morphological Parts of *Ligustrum vulgare*

**DOI:** 10.3390/molecules29020399

**Published:** 2024-01-13

**Authors:** Szymon Litewski, Izabela Koss-Mikołajczyk, Barbara Kusznierewicz

**Affiliations:** Department of Chemistry, Technology and Biotechnology of Food, Faculty of Chemistry, Gdańsk University of Technology, 11/12 Narutowicza St., 80-233 Gdańsk, Poland; szymon.litewski@pg.edu.pl (S.L.); izabela.koss-mikolajczyk@pg.edu.pl (I.K.-M.)

**Keywords:** *Ligustrum vulgare*, UHPLC, HRMS, oleuropein, antioxidant activity, cyclooxygenase-2 inhibition, α-amylase inhibition, cytotoxicity

## Abstract

*Ligustrum vulgare* (LV), widely cultivated in Europe and often used in hedges, has been historically recognized in folk medicine for its potential health benefits. This study focused on exploring the untargeted identification of secondary metabolites in ethanol extracts (70% *v*/*v*) from different morphological parts (young shoots, leaves, flowers and fruits) of LV at various stages of plant development, using ultra-high-performance liquid chromatography with high-resolution mass spectrometry (UHPLC-HRMS). Additionally, the selected biological activities (antioxidant activity, cyclooxygenase-2 inhibition (COX-2), α-amylase inhibition and cytotoxicity) of the tested extracts were determined. Untargeted metabolomics showed that LV extracts were a rich source of phenylethanoid compounds, flavonoids, iridoids and their derivatives. The flowers of LV had the highest content of oleuropein (33.43 ± 2.48 mg/g d.w.). The lowest antioxidant activity was obtained for ripe and post-seasonal fruits, while in the case of other samples, the activity was at a similar level. All tested extracts showed α-amylase and COX-2 inhibitory activity. In addition, LV extracts showed strong antiproliferative properties in colorectal (HT29) and liver (HepG2) cancer cell lines. The obtained results show the difference in the content of bioactive compounds in various morphological parts of *Ligustrum vulgare*. These differences may influence the multifaceted medicinal potential of this plant.

## 1. Introduction

Plants from the *Oleaceae* family have been known for their medicinal properties for thousands of years. In both Chinese and Mediterranean folk medicine, these plants were used in the prevention of many noncommunicable diseases such as cardiovascular diseases, chronic inflammation, hypertension, type II diabetes and cancer [1]. The main bioactive phytochemicals present in *Oleaceae* plants are iridoids, and one of the best known is oleuropein, which was reported in the genera of *Olea*, *Ligustrum*, *Syringa*, *Osmanthus*, *Jasmonium*, *Fraxinus*, *Phillyrea* and *Forestiera* [2]. Over the last few decades, numerous studies have demonstrated the bioactive effects of oleuropein, most of which are directly linked to its strong antioxidant activity [3]. The presence of oleuropein in olives (*O. europaea*) also contributes to the positive impact of extra virgin olive oil consumption on overall health [4].

The genus *Ligustrum* L. (*Oleaceae*) includes about 40 species of evergreen or deciduous shrubs found mainly in Asia. There are only two representatives of the genus *Ligustrum* in Europe: the common privet (*L. vulgare*) and the ovoid-leaved privet (*L. ovalifolium*). The first is a native species, growing wild or cultivated as part of hedges, while the second is an invasive species. Common privet is closely related to the European species of lilac (*S. vulgaris*), and the similarity between them can be observed in the structure of the inflorescence with characteristic four-lobed, spherical crown lobes [5].

Plants from *Ligustrum* L. are widely known for their health-promoting properties. In the last decade, a bitter-tasting herbal tea called *Ku-Ding-Cha*, known in southwestern China since 200 BC, has gained great popularity around the world, and among its ingredients are the dried leaves of various *Ligustrum* species, primarily *L. robustum*, but also *L. purpurascens*, *L. henryi*, *L. lucidum*, *L. sinense* and *L. japonicum*. This tea is believed to support weight loss, prevent diabetes, prevent high blood pressure and reduce inflammation [6,7]. Another common product in Chinese medicine is dried, ripe fruits of *L. lucidum*, called *nüzhenzi*. These raisin-like fruits are not only used as herbal medicine but have also been approved by the China Food and Drug Administration (CFDA) as a health-promoting functional food. The first mention of *nüzhenzi* appeared in “Shennong-Bencao-Jing”, the oldest known book of Chinese medicine on natural raw materials. According to beliefs, their main role is to nourish the liver and kidneys and strengthen the bones. Thanks to modern science, it is known that *L. lucidum* fruit extracts have hepatoprotective, anticancer, anti-inflammatory, antioxidant and anti-osteoporosis properties [8].

Despite the prevalence of *L. vulgare* in Europe, its use in folk medicine is known only in the Mediterranean region. The medicinal properties of the *L. vulgare* leaves were described in the 1st century B.C. by the Greek pharmacologist Dioscorides [9]. Chewing fresh privet leaves was believed to soothe inflammation of the mouth, and a decoction of the above-ground parts of the plant was used against burns and headaches. Meanwhile, fruit juice was used to clean wounds. In addition, in the 18th century, the leaves were attributed to properties against diarrhea and scurvy [10]. Nowadays, in Latium (Italy) and Western Anatolia (Turkey), fresh leaves are used as a remedy for canker sores. In Cyprus, the plant is attributed to antirheumatic properties, while in Azerbaijan, it is used as a remedy for hypertension [10,11]. On the other hand, in the rest of Europe, *L. vulgare* is widely recognized as a poisonous plant, but data on this subject are conflicting. There have been documented cases of people who, after consuming privet fruit, developed digestive system complaints such as nausea, diarrhea, vomiting and abdominal pain. Moreover, it is believed that the fruit may be acutely toxic, especially for children [12]. However, the substance responsible for the described ailments has not been identified; thus, the toxic dose for humans has not been determined. 

Several studies described the phytochemical composition of *L. vulgare* extracts, where the main compounds were flavonoids, secoiridoids and phenylethanoids [13,14,15]. The oleoside-type secoiridoids are associated with various biological activities that have been extensively studied in recent years. Particularly important is the antioxidant activity, which is demonstrated primarily by secoiridoid derivatives with a hydroxytyrosol moiety [16]. Antioxidants have proven influence on the occurrence of cardiovascular diseases, inflammation and diabetes. The results of our previous study showed significant radical scavenging activity of *L. vulgare* extracts, where the detected antioxidants were mostly from the group of iridoids, including oleuropein and its derivatives [17].

Due to the growing interest in traditional medicine, there is still a need for phytochemical and pharmacological research that would not only expand the existing therapeutic potential of *Ligustrum* species but also determine the safety of its use. However, research to date has mainly focused on leaves or fruits. In this study, a detailed metabolomic analysis coupled with multivariate data analysis of extracts obtained from various morphological parts of privet (young shoots, leaves, flowers and fruits) of various degrees of ripeness was performed for the first time. The aim of this study was to determine the potential of common privet (*Ligustrum vulgare*) as a source material for obtaining bioactive substances, including oleuropein. In addition, antioxidant activity (ABTS, DPPH), anti-inflammatory activity (COX-2 inhibition) and antiproliferative activity in two cancer cell lines (HepG2 and HT29) were determined. Moreover, a novelty method for determining the antidiabetic activity (α-amylase inhibition) using high-performance thin-layer chromatography (HPTLC) was used. 

## 2. Results and Discussion

### 2.1. Metabolomic Analysis of L. vulgare Extracts

**Figure 1:**
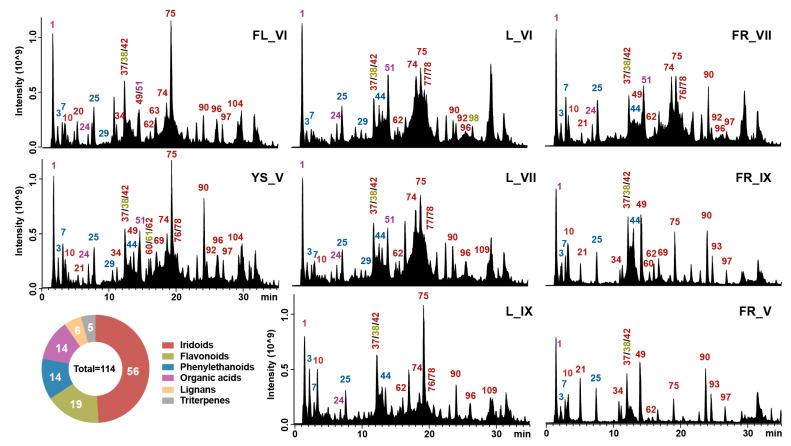
TIC of metabolite profiles of *Ligustrum vulgare* extracts acquired in ESI(−) set with number of metabolite classes (pie chart) annotated in these samples. The abbreviations: FL, YS, L and FR refer to the morphological part of *Ligustrum vulgare* and mean, respectively: flowers, young shoots, leaves and fruits, while the numbers IV-IX indicate the month of their harvest. The peak numbers correspond to the compound numbers in Table 1.

The untargeted metabolomic analysis processed by UHPLC-HRMS with Compound Discoverer 3.3 software was used to identify the wide range of compounds present in the extracts of different morphological parts of *Ligustrum vulgare* (LV) collected from May to September 2022. In this research, the majority of the substances exhibited a stronger reaction when analyzed in the negative mode as opposed to the positive mode. Figure 1 displays examples of the total ion chromatograms (TICs) of the LV extracts studied. The compounds were identified by comparing their retention times and mass spectra, as obtained from Orbitrap-HESI-MS, with those of authenticated standards where possible. For the remaining compounds for which commercial standards were not available, their characterization relied on the interpretation of their mass spectra, Human Metabolome and PubChem data and information previously documented in the existing literature. The analysis permitted annotation of 114 metabolites in LV extracts, spanning over six major classes (Figure 1, pie chart). They comprised 56 iridoids, 19 flavonoids, 14 phenylethanoids, 14 organic acids and derivatives, 6 lignans and 5 triterpenes. Table 1 summarizes the alleged identification of these phytochemicals.

In the group of organic acids and derivatives, 14 compounds were tentatively identified. The main compound from this group found in LV extracts was D-(−)quinic acid (**1**) with a pseudo-molecular ion at *m*/*z* 191.055. Additionally, five esters of quinic acid were detected; notably, compounds (**6**) and (**16**), with a parent ion at *m*/*z* 353.088, shared the same MS fragmentation pattern with ions at *m*/*z* 179.034, 161.023 and 137.023, characteristic of the caffeic acid moiety, and were thus tentatively assigned as two caffeoylquinic acid isomers. Compound **24** with a quasi-molecular ion at *m*/*z* 337.093 was identified as *p*-coumaroylquinic acid by comparing the fragmentation pattern with the literature data. The mass of compound **51**, corresponding to a deprotonated molecular ion at *m*/*z* 321.097, was lower by 16 Da than the mass of *p*-coumaroylquinic acid, which indicates the lack of a hydroxyl moiety. Moreover, the presence of an MS/MS fragment at *m*/*z* 147.044 could correspond to a cinnamoyl moiety. Thus, compound **51** was tentatively assigned as cinnamoylquinic acid. Compound **26** was initially identified as feruloylquinic acid. According to Kuhnert et al. [24], the regioisomers of feruloylquinic acid can be identified by the intensity of the base and secondary peak. In this study, the main MS^2^ fragments at *m*/*z* 173.044, 134.036 and 193.050 suggest that compound **26** could be 1-isoferuloylquinic acid or 4-feruloylquinic acid. Based on the presence of MS^2^ fragments at *m*/*z* 91.054 and 65.038 with a precursor ion at *m*/*z* 163.039, compound (**30**) was identified as *p*-coumaric acid. Moreover, compounds (**15**) and (**18**) that gave ion [M − H]^−^ at *m*/*z* 325.093 were assigned as *p*-coumaric acid hexosides, as they differed 162 Da from compound (30), corresponding to the hexoside moiety. Similarly, compound (**13**) was identified as *p*-coumaroylhexaric acid. These compounds (**13**, **15**, **18**) were reported in the *Oleaceae* family previously [21,22]. Compound (**17**) with a precursor ion at *m*/*z* 151.039 and a major fragment at *m*/*z* 123.044 as a result of the loss of the CHO group (38 Da) was identified as vanillin. Compound (**2**) was assigned as citric acid. Moreover, the pseudo-molecular ion of compound (**12**) was observed as an adduct with formic acid [M – H + FA]^−^ (*m*/*z* 417.140), followed by fragments at *m*/*z* 209.081, 194.058 and 176.057. This characteristic fragmentation pattern was described previously in the literature, which allowed for the identification of compound (**12**) as syringin [20]. Finally, the coumarin derivative (**22**) has been identified in *L. vulgare* extracts, namely esculetin ([M − H]^−^ at *m*/*z* 177.018), with a characteristic MS^2^ fragment at *m*/*z* 133.028, indicating CO_2_ loss. Eculetin was previously isolated from the leaves and bark of the *Olea europaea*, so it may also occur in other members of the *Oleaceae* family [19].

Phenylethanoids (PhEs) are another important group of secondary metabolites of *Oleaceae* plants. In this study, 14 PhEs were identified. The main compound from this group was hydroxytyrosol (**9**), which was identified based on the molecular ion at *m*/*z* 153.054 and the MS^2^ fragment at *m*/*z* 123.044, corresponding to the loss of CH_2_OH moiety. Additionally, hydroxytyrosol glucoside (**3**) (+162 Da) and hydroxytyrosol acetate (**40**) (+46 Da) were identified. Compound (**4**) was detected using the ion [M − H]^−^ at *m*/*z* 461.166 and MS^2^ fragment at 315.108, which indicated an additional hexose residue in hydroxytyrosol glucoside. Thus, compound (**4**) was identified as bioside. The identification of salidroside (**7**), which is tyrosol glucoside, was based on the characteristic fragmentation ion at *m*/*z* 137.059, corresponding to tyrosol. Moreover, salidroside in combination with the apiofuranosyl residue (+162 Da) forms osmanthuside H (**8**) ([M − H]^−^ at *m*/*z* 431.155), which was also detected. Compound (**25**) with the [M − H]^−^ ion at *m*/*z* 785.251 that eluted at 7.50 min was accurately identified as echinacoside in comparison with a standard. Additionally, based on the presence of fragment ions derived from caffeic acid (*m*/*z* 161, 135, 179), three isomers of verbascoside (**44**, **56**, **59**) ([M − H]^−^ at *m*/*z* 623.198), an isomer of echinacoside (**29**) ([M − H]^−^ at *m*/*z* 785.251), syringalide A (**68**) ([M − H]^−^ at *m*/*z* 461.145) and two isomers of isosyringalide rhamnoside (**65**, **71**) ([M − H]^−^ at *m*/*z* 607.203) were tentatively identified.

Flavonoids are largely distributed in plants. In this study, 20 flavonoids were identified in aqueous-ethanolic extracts of *L. vulgare*, mainly flavonols, flavanones, flavones and anthocyanins. Compound (**38**) was easily identified as quercetin-3*-O-*rutinoside (rutin) by comparison of the retention time and MS spectrum with the authentic standard. Compound (**14**) with a pseudo-molecular ion at *m*/*z* 593.151 was assigned as cyanidin-3*-O-*rutinoside. The MS/MS spectra of flavones showed a typical fragment at *m*/*z* 269.045, indicating the presence of *O-*glycoside (**70**) (+162 Da) and *O-*rutinoside (**61**, **66**) (+308 Da) forms of apigenin (**107**). The precursor ion [M − H]^−^ at *m*/*z* 285.040 of compound (**98**) could be assigned as kaempferol or luteolin aglycone; however, their glycosides differ in elution time. These flavonoids were identified based on the literature data, which indicate that these glycosides should elute in the order luteolin-7*-O-*rutinoside (**39**), keampferol-7*-O-*glucoside (**46**) and kaempferol-3*-O-*rutinoside (**47**) [18]. Compound (**32**) with an [M − H]^−^ ion at *m*/*z* 609.146 and a fragment ion at *m*/*z* 285.040 eluted at 10.69 min was identified as luteolin diglucoside, as its mass differs from typical luteolin by 324 Da. Moreover, compound (**58**) was identified as luteolin-7*-O-*rhamnoside. Compound (**53**), having an additional two rutinoside moieties (284 Da) with a fragment ion derived from apigenin (*m*/*z* 269.045), was identified as ligustroflavone, previously isolated from LV [7,13]. Compound (**73**) with a precursor ion [M − H]^−^ at *m*/*z* 461.109 was proposed as chrysoeriol-7*-O-*glucoside, while compound (**11**) ([M − H]^−^ at *m*/*z* 465.104) was tentatively identified as a taxifolin-3*-O-*glucoside. Compound (**106**) ([M − H]^−^ at *m*/*z* 271.061) with characteristic MS^2^ fragmentation ions at *m*/*z* 151.002, 119.049 and 107.012 was tentatively identified as a naringenin as well as its hexoside form (**64**). Another flavonone identified in both glycoside (**36**) and aglycone form (**91**) was eriodicytol, also found in *Oleaceae* previously [23].

In the tested LV extracts, 56 iridoids were identified, which is more than half of the total number of identified compounds. Iridoids are considered a chemotaxonomic marker used in the identification of members of the *Oleaceae* family [2]. Compound (**10**) was detected at *m*/*z* 389.109, and as a result of the loss of the hexoside (−162 Da) and C_2_H_2_O_5_ (−106 Da), a fragment ion at *m*/*z* 121.064 was formed, which indicated the typical fragmentation pattern for oleoside. Additionally, two isomers of oleoside 11-methyl ester (**21**, **23**) could be tentatively identified by the pseudo-molecular ion [M − H]^−^ at *m*/*z* 403.124. Based on the presence of an MS^2^ signal at *m*/*z* 139.002 and the deprotonated ion [M − H]^−^ at *m*/*z* 241.071, compound (**27**) was tentatively assigned as elenolic acid. In the case of peak (**28**), based on the [M − H]^−^ at *m*/*z* 225.076 and the specific fragmentation pattern (*m*/*z* 123.044, 101.023, 68.997) according to PubChem, this compound was tentatively identified as genipin. The mass of the compound (**20**) was 324 Da greater than compound (**28**), which could indicate the presence of two hexoside residues. Thus, compound (**20**) was tentatively characterized as genipin 1-gentiobioside. Genipin is a characteristic monoterpene iridoid isolated previously from fruits of *Gardenia jasmonides* and *Genipa americana* [29]. Therefore, both compounds (**28**) and (**20**) were reported in the *Ligustrum* genus for the first time. Compound (**75**) was easily identified as oleuropein ([M − H]^−^ at *m*/*z* 539.177) by comparison with the standard. Additionally, three oleuropein isomers (**80**, **102**, **105**) were also identified based on the characteristic oleuropein MS/MS fragments at *m*/*z* 307.082, 275.093, 149.023 and 139.039. Compounds (**85**), (**87**), (**96**), (**100**) and (**109**) were tentatively assigned as oleuropein aglycones, with the pseudo-molecular ion at *m*/*z* 377.124 corresponding to the loss of glucose (−162 Da). Moreover, oleuropein derivatives were also present in LV extracts, namely oleuropeinic acid (**35**) ([M − H]^−^ at *m*/*z* 569.151) and oleuropein dihexoside (**52**) ([M − H]^−^ *m*/*z* 701.230). Compounds (**37**), (**42**), (**45**) and (**103**), assigned as 10-hydroxyoleuropein, exhibited a deprotonated molecular ion at *m*/*z* 555.171 with a fragment ion at *m*/*z* 307.083. This suggests that the hydroxyl group was combined with the oleoside moiety. Similarly, compound (**31**) was tentatively identified as 7-hydroxyoleuropein; despite the pseudo-molecular ion having the same mass of (*m*/*z* 555.171) as 10-hydroxyoleuropein, the fragmentation pattern was different. Moreover, five isomers of oleacein (**72**, **74**, **76**, **77**, **78**) ([M − H]^−^ at *m*/*z* 319.118) were also identified. Due to the similar retention time to oleuropein, oleacein is probably a product of its degradation. However, oleacein had been isolated from *L. vulgare* previously [30]. Compounds (**90**) and (**95**) were detected because of [M − H]^−^ at *m*/*z* 523.182, with fragments at *m*/*z* 361.129, 291.087 and 259.097 corresponding to the successive loss of the glucose C_4_H_6_O and CH_3_OH, respectively. The mass of these fragments was 16 Da less than those of oleuropein, which indicates that compounds (**90**) and (**95**) were ligstroside isomers. Additionally, two isomers of ligstroside aglycone (**99**, **108**) were also tentatively identified. As the pseudo-molecular ions of compounds (**62**) and (**69**) (*m*/*z* 539.177) were the same as in the case of oleuropein, their characteristic MS^2^ fragment at *m*/*z* 291.087 indicates the presence of a hydroxyl group at the methyloleoside moiety. Thus, compounds (**62**) and (**69**) were tentatively identified as 10-hydroxyligstroside isomers as well as their aglycone forms (**88**, **92**). Other iridoid compounds were detected by the [M − H]^−^ at *m*/*z* 685.235 with the main MS^2^ fragments at *m*/*z* 453.140, 299.113 and 223.061, which are specific for nuzhenide and its isomers (**41**, **49**, **50**). The mass of compound (**82**) ([M − H]^−^ at *m*/*z* 523.182) suggested the presence of another ligstroside isomer; however, the MS/MS spectra showed a fragment at *m*/*z* 453.141, characteristic of nuzhenide. Additionally, a fragment at *m*/*z* 137.060, corresponding to the loss of tyrosol residue, was detected. Thus, based on this information, compound (**82**) was tentatively assigned as nuzhenide aglycone. Compounds (**79**), (**93**) and (**97**) were tentatively identified as GL3 or oleonezuhine. These compounds shared the same MS/MS fragments (*m*/*z* 1071.355, 909.303, 685.235), which indicates that these compounds were indeed nuzhenide derivatives with an additional oleoside 11-methylester moiety. Compounds (**34**), (**43**) and (**57**) ([M − H]^−^ at *m*/*z* 701.230) showed an MS^2^ signal at *m*/*z* 315.108, corresponding to the hydroxytyrosol glucoside moiety. Thus, these compounds were tentatively identified as an isomer of neonuzhenide, which is nuzhenide with an additional hydroxytyrosol moiety. Compounds (**63**), (**81**) and (**86**) were assigned as comselogoside isomers due to the presence of the [M − H]^−^ ion at *m*/*z* 535.146 and the characteristic fragments produced by the loss of a coumaroyl moiety (*m*/*z* 163.039, 145.028). Similarly, compound (**67**) was tentatively identified as caffeoyl-6′-secologanosie based on the presence of the caffeoyl fragments (*m*/*z* 179.034, 161.023). Compounds (**89**) ([M − H]^−^ at *m*/*z* 685.235) and (**101**) ([M − H]^−^ at *m*/*z* 925.298) were tentatively identified as excelside B and jaspolyoside, respectively. Compound (**83**) was identified as oleoactoside, which is a complex of verbascoside with an oleoside moiety, previously found in *Osmanthus fragrans* (*Oleaceae*) [25]. Compound (**84**) with [M − H]^−^ at *m*/*z* 583.203 was identified as lucidomoside C. Finally, compounds (**94**) and (**104**) that shared the same fragmentation pattern (*m*/*z* 161.060, 147.044, 121.064) were identified as 6′*-O-trans*-cinnamoyl-8-epikingisidic acid and 6′*-O-cis*-cinnamoyl-8-epikingisidic acid, respectively. Both were reported in the *L. lucidum* fruits where the *cis* isomer eluted later than the *trans* isomer [18].

Another group of compounds identified in this study were lignans. Compound (**33**), assigned as lariciresinol-4*-O-*glucoside, was detected at *m*/*z* 521.202, with the main MS/MS fragment at *m*/*z* 329.139 corresponding to the loss of glucose and CH_2_O residue. Compound (**54**) with a deprotonated molecular ion at *m*/*z* 579.208 was tentatively identified as syringaresinol*-O-*glucoside. The MS^2^ ion fragment at *m*/*z* 417.155 was attributed to the loss of a hexoside moiety. Another lignan was found at *m*/*z* 519.187, which was assigned as pinoresinol-4*-O-*glucoside (**48**). The MS/MS fragments of compound (**55**) (*m*/*z* 387.145, 372.121) were 30 Da greater than the characteristic fragmentation pattern of pinoresinol-4*-O-*glucoside (*m*/*z* 357.134, 342.110), indicating the presence of a methoxy group; thus, compound (**55**) was assigned as medioresinol-4*-O-*glucopyranoside. Compounds (**5**) and (**19**) were identified as acyclodihydroelonolic acid and cycloolivil glucoside, respectively. According to Toth et al. [23], both compounds were detected in olive wood and leaves previously.

In addition to the above-mentioned compounds, a total of five triterpenoids were also found in LV extracts. Compound (**113**) was tentatively characterized as ursolic acid or oleanolic acid since they both could generate the same pseudo-molecular ion [M − H]^−^ at *m*/*z* 455.353. Additionally, the masses of compounds (**111**) (*m*/*z* 471.348) and (**110**) (*m*/*z* 487.343) were, respectively, 16 Da and 32 Da greater than the mass of compound (**113**). Thus, compound (**111**) was identified as colosis acid (2α-hydroxyursolic/olenolic acid), while compound (**110**) was assigned as tormentic acid (2α’,19α-dihydroxyursolic acid). Similarly, acetyloleanolic/ursolic acid (**114**) was detected by a precursor ion [M − H]^−^ at *m*/*z* 497.363. Finally, the MS^2^ fragment at *m*/*z* 145.028 in the MS spectrum of compound (**112**) indicates the presence of coumaroyl residue; thus, this compound was identified as 3β*-O-cis*/*trans*-*p*-coumaroylmaslinic acid. All the triterpenoids detected in this study were reported in the fruits of *L. lucidum* previously [18].

### 2.2. Comparative Analyses of Phytochemicals in L. vulgare Extracts

**Figure 2:**
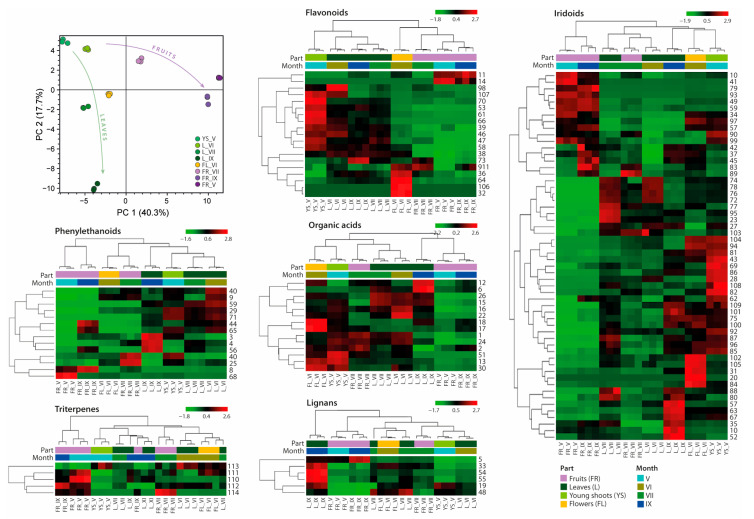
Metabolic changes of extracts from different parts of *Ligustrum vulgare* harvested in different periods presented as a PCA score plot and heatmaps. The abbreviations: FL, YS, L and FR refer to the morphological part of *Ligustrum vulgare* and mean, respectively: flowers, young shoots, leaves and fruits, while the numbers IV-IX indicate the month of their harvest. The row numbers in heatmaps correspond to the compound numbers in Table 1.

The distribution of individual secondary metabolites depends on the morphological part and stage of maturity of the plant. Untargeted metabolomics analysis generates vast data on the complex number of metabolites that must be managed by powerful statistical tools. Thus, for the comparison of the studied samples, principal component analysis (PCA) was performed. In the case of PCA, the total variance of 90.9% was explained by the five PCs in the model; meanwhile, the first two principal components (PC1 and PC2) contributed 40.3% and 17.7% to the total score, respectively. The result presented in Figure 2 showed that the phytochemical composition of ripe and post-seasonal fruits exhibits clear separation from compounds obtained from other morphological parts. However, in the PC2, the LV leaves collected in September differ from the other studied samples. Based on the PCA models (Figure 2) and obtained chromatograms (Figure 1), the results indicate that the wintering of fruit does not significantly affect the chemical composition of ripe fruit, but changes are especially noticeable when comparing young fruits with ripe ones.

To provide a more detailed comparative analysis of the chemical composition of individual extracts, a cluster hierarchy analysis was performed for the identified 114 compounds. In Figure 2, the data represent a deviation from the average content of compounds shown as heatmaps of individual groups based on peak areas in the mass spectrum. The black color represents the average content of a given compound in the tested extract. Therefore, the higher content of the compound in comparison to the average is represented in red, while the lower content is in green. It should be noted that the analysis was carried out for each compound separately but not for the entire group of individual metabolites. However, to simplify the illustration, individual heatmaps corresponding to groups of metabolites have been divided into sections. In addition, the relationships between the studied LV extracts as well as their phytochemical composition are presented as a cluster. It was observed that in most cases, the phytochemical composition of ripe and post-seasonal fruits is constant.

Based on the heatmap of the content of individual organic acids and derivatives, it was noticed that this group of compounds does not apply to ripe fruits. The highest content of organic acids was found in flowers and young shoots of LV. Quinic acid and its derivatives were found mainly in leaves and young shoots of privet, while in mature fruits, they were present in trace amounts. September leaves were the main source of syringin (**12**), also known as ligustrin, which is generally considered toxic.

The heatmap indicates that June leaves were the main source of a wide range of phenylethanoids (**40**, **9**, **59**, **29**, **71**, **44**, **65**). A slightly lower average content of these compounds occurred in the remaining leaves. However, in the case of September leaves, the main compounds were hydroxytyrosol glucoside, bioside and verbascoside, which are structural derivatives. In addition, due to several biological properties of verbascoside, ripe fruits of *L. vulgare* also seem to be an interesting source of this compound. The only two phenylethanoids found in post-seasonal fruits were syringalide A (**68**) and osmanthuside H (**8**). Echinacoside (**25**) was the main compound in unripe fruits, which is consistent with the peak intensity in the chromatogram (Figure 1), while the presence of the formed hydroxytyrosol acetate (**40**) may result from the degradation of oleuropein. The formation of this compound was observed in the case of metabolomic analysis of European olive fruit during the olive oil pressing process [22].

In the case of flavonoids, an interesting relationship was observed between the occurrence of individual compounds and the morphological part of LV. Namely, two clusters were formed that separate the green parts of the plant from those directly related to fruiting. Among the green parts, most of the identified flavonoids occurred in young shoots, while in the leaves, they remained at an average level. The exception was luteolin (**98**), the content of which was the highest in June leaves after which its content gradually decreased in the following months. Moreover, among the flavonoids, the main compound present at a similar level mostly in leaves of *L. vulgare* was quercitin-3′-*O*-rutinoside (**38**). The presence of naringenin (**106**) and luteolin-4′,7-*O*-diglucoside (**32**) was limited to LV flowers only. Cyanidin-3-*O*-rutinoside (**14**) is the main anthocyanin in black olive fruits, indicated by their purple color [27]. Therefore, this compound was found only in ripe fruits of *L. vulgare*.

Among a wide range of iridoids, the main compounds present in the mature fruits (FR_IX and FR_V) of *L. vulgare* were derivatives of nuzhenide, namely oleonuzhenide and neonuzhenide, GL3. In reference to the research of Li et al. [18], all these compounds are the main components of *L. lucidum* fruits, also called nüzhenzi. Due to their numerous health-promoting properties, these fruits have been known in Chinese medicine for centuries. Additionally, in the case of ripe fruits (FR_IX), the average content of hydroxyoleuropein isomers (**37**, **42**, **45**) was significantly higher than in the case of the other tested samples. The presented heatmap strongly highlights the significantly higher average iridoid content in young shoots, while this level changes as the leaves mature. Oleacein plays a key role in oleuropein biosynthesis. According to Obied et al. [31], the content of oleuropein increases during fruiting, while in the case of oleacein, there is an inversely proportional relationship, which was also observed in this study. The fruiting period of LV falls in July and the highest amount of oleacein (**72**, **74**, **76**, **77**, **78**) was observed in leaves collected during this period. Although ligstoside (**90**) may also be a precursor in oleuropein biosynthesis, it was mostly detected in the case of young shoots and mature fruits. On the other hand, ripe fruits had the lowest oleuropein content. Moreover, the characteristic iridoids occurring primarily in flowers were two isomers of oleuropein (**102**, **105**), 7-hydroxyoleuropein (**31**) and genipin-1-gentiobioside (**20**).

Among the small group of lignans, most of them were found mainly in September leaves, while compounds from the triterpene group occurred mainly in ripe fruits. The content of acetyloursolic acid (**114**) was limited only to fruits. 

### 2.3. Quantitative Analysis of Selected Phytochemicals in L. vulgare Extracts

To determine the concentration of the most present compounds in LV extracts, the UHPLC-DAD method was used. Oleuropein, rutin and echinacoside were selected for quantitative determination, and the results obtained are presented in Table 2. The results of the statistical analysis (one-way ANOVA and Tukey’s post-test) of obtained data are attached as Supplementary Materials (Tables S1–S3). Extracts from *L. vulgare* flowers and September leaves were the richest source of oleuropein with a content of 33.43 ± 2.48 and 33.20 ± 1.83 mg/g of dry matter, respectively. The content of oleuropein in olive leaves is, on average, 60–90 mg/g of dry matter [32]. Considering the fact that common privet has less demanding growing conditions than *Olea europaea*, LV leaves and flowers can be considered a profitable source of oleuropein. In the case of June and July leaves, as well as between July leaves and fruits collected at the same time (FR_VII), the differences in the content of oleuropein were not significant (*p* > 0.05). However, the ripe fruit extracts had approximately ten times less oleuropein than the extract from September leaves that were collected at the same time. According to Ranalli et al. [33], the concentration of oleuropein decreases as the olives mature, which was similarly determined in the case of *L. vulgare* fruits. Thus, trace amounts of oleuropein were detected in the extract from post-seasonal fruits (0.67 ± 0.23 mg/g d.w.) [32]. The echinacoside content was highest in young fruits (28.88 ± 0.21 mg/g d.w.), while its content gradually decreased during leaf development. There was no significant difference (*p* > 0.05) in the echinacoside content between extracts of June flowers and leaves and between post-seasonal fruits (FR_V) and September leaves. However, according to the study performed by Czerwinska et al. [34], the content of echinacoside increased in the leaves of *L. vulgare* from May to September. The discrepancy in the results obtained may be due to many factors that influence the biosynthesis of secondary metabolites. Moreover, the rutin content in privet leaves increased from May to September in the range from 9.54 ± 0.26 mg/g d.w. to 13.75 ± 0.96 mg/g d.w., while in the case of fruits, the highest rutin content was in ripe fruits (6.08 ± 0.58 mg/g d.w.).

### 2.4. Antioxidant Activity of L. vulgare Extracts

**Figure 3:**
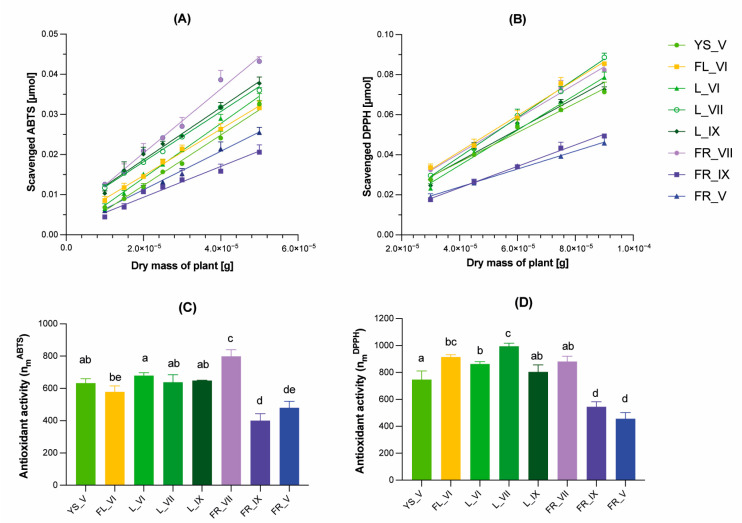
Antioxidant activity of extracts from different morphological parts of *Ligustrum vulgare* determined by spectrophotometric tests with ABTS (**A**,**C**) and DPPH (**B**,**D**) radicals. The results are means ± SD of three independent determinations. The bar graphs show total antioxidant activity expressed as coefficients: n*_m_^ABTS^*- μmoles of ABTS reduced by compounds derived from 1 g of lyophilizates (**C**), n*_m_^DPPH^*- μmoles of DPPH reduced by compounds derived from 1 g of lyophilizates (**D**). The abbreviations used: FL, YS, L and FR refer to the morphological part of *Ligustrum vulgare* and mean, respectively: flowers, young shoots, leaves and fruits, while the numbers IV-IX indicate the month of their harvest. Bars marked with the same letters indicate values that are not significantly different according to one-way ANOVA with Tukey’s multiple comparison test at *p* ≤ 0.05.

Our previous study showed that the profile of antioxidant compounds of aqueous-ethanolic extracts of *L. vulgare* depends not only on the morphological part of the plant but also on the maturity stage of the plant sample [17]. In the current study, the total antioxidant activity determination was performed using two standard spectrophotometric methods (ABTS and DPPH) but with some modifications. Typically, the total antioxidant activity is presented as the Trolox equivalent antioxidant capacity (TEAC) or EC50 value, which indicates the concentration needed to scavenge 50% of free radicals (ABTS or DPPH). However, these parameters are strongly dependent on the concentration of radical used and require the use of standards (Trolox). Therefore, the protocols of standard spectrophotometric tests (ABTS and DPPH) used in this study were modified to show the kinetic aspect of redox reactions. This approach was performed previously for pure standards [35,36,37] and for the plant samples [38,39]. Therefore, the antioxidant activity was expressed as the stoichiometric n10 value that describes how many molecules of a radical were scavenged by 1 g of plant dry mass within a 10 min reaction time. Statistical analysis of the obtained results (one-way ANOVA with Tukey’s multiple comparison test) is attached in the Supplementary Materials (Tables S4 and S5). As shown in Figure 3, the studied LV extracts tended to be better scavengers of DPPH than of ABTS radicals. In the ABTS test, the strongest antioxidant potential among the tested extracts showed unripe fruits (FR_VII), followed by leaves, young shoots and flower extracts, while the ripe and post-seasonal fruits showed the lowest antioxidant potential of all tested samples. A similar trend can be observed in the DPPH test, where extracts from leaves, flowers, young shoots and unripe fruits showed similarly strong antioxidant potential, while ripe and post-seasonal fruits were characterized by the lowest activity. It can be explained by the results of UHPLC analysis (Figure 2), which showed that post-seasonal and ripe fruit (FR_V and FR_IX) extracts compared to other tested samples are characterized by a low content of phenolic acids and flavonoids, that these compounds are mainly responsible for the antioxidant potential of the tested extracts. The results of total antioxidant activity are consistent with previous results of antioxidant profiling, which generally showed a lower content of compounds with radical scavenging ability in ripe fruit extracts compared to other parts of *L. vulgare* [17].

### 2.5. α-Amylase Inhibitory Activity of L. vulgare Extracts

Type 2 diabetes is one of the most widespread metabolic diseases in the world and poses a serious health risk. One of the basic strategies used in the treatment of hyperglycemia is the use of amylolytic enzyme inhibitors. In this study, in order to determine the α-amylase inhibitory activity, an innovative new method was used that involves chromatographic separation of starch decomposition products by a given enzyme and then densitometric detection of individual analytes. α-Amylase is an enzyme that hydrolyzes polysaccharides containing three or more D-glucose molecules connected by glycosidic bonds at the α-1,4 positions. The main products resulting from the breakdown of starch by α-amylase are maltose and dextrins [40]. Therefore, the degree of inhibition of a given enzyme by the tested LV extracts was determined by comparing the intensity of the bands corresponding to maltose, which was formed in the presence of the extracts (as potential inhibitors) with the intensity of the band created in the control mixture without inhibitor. Unlike typical spectrophotometric methods commonly used to determine α-amylase activity, the approach proposed in this work enables quantitative tracking of all enzymatic decomposition products arising from the substrate and not their total content, as is the case in spectrophotometric tests. 

**Figure 4:**
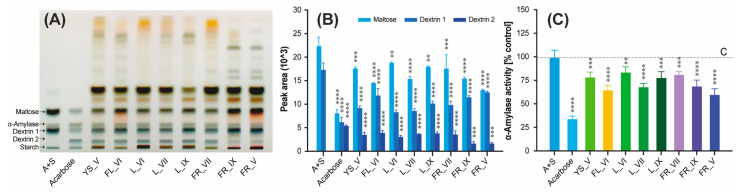
α-Amylase inhibitory activity of *Ligustrum vulgare* extracts determined using HPTLC method. Panel (**A**) shows profiles of starch decomposition products obtained in starch and α-amylase mixtures without inhibitors (A + S; track 1) or with inhibitors such as acarbose (track 2) or the tested LV extracts (tracks 3–10). Panel (**B**) shows the content of individual starch decomposition products (maltose and dextrins) present in tested mixtures calculated based on the densitograms from HPTLC chromatogram. Panel (**C**) shows α-amylase activity calculated based on the amount of maltose present in tested mixtures compared with the control mixture without inhibitor. The abbreviations used: A + S refer to the positive control, FL, YS, L and FR refer to the morphological part of *Ligustrum vulgare* and mean, respectively: flowers, young shoots, leaves and fruits, while the numbers IV-IX indicate the month of their harvest. Results represent means ± SD. Significantly different values determined by using one-way analysis of variance (ANOVA) with Dunnet’s post-test are marked as ** *p* < 0.01; *** *p* < 0.001; **** *p* < 0.0001.

The profiles of products of the enzymatic decomposition of starch in the presence of LV extracts obtained using the HPTLC technique are presented in Figure 4A. Both maltose and dextrin bands were observed in all reaction mixtures, which proves the catalytic activity of α-amylase. However, the bands (dextrin 1, dextrin 2 and maltose) obtained as a result of the reaction with the known inhibitor, acarbose (second track,) are much less intense than in the case of the control sample (first track), which proves the inhibition of the enzyme. 

In order to compare the degree of *α*-amylase inhibition from the obtained chromatogram (Figure 4A), densitograms were generated for all reaction mixtures. Then, on the basis of the peak areas, the approximate content of individual starch decomposition products was determined. The obtained results are presented in Figure 4B, while statistical analyses of the obtained results using one-way ANOVA with Tukey’s multiple comparison test are attached as Supplementary Materials (Tables S6–S8). It is clear that all tested plant extracts of LV show an inhibitory effect on α-amylase, as the maltose and dextrin 1 peak areas decreased in comparison to the control sample. Also, an additional peak from dextrin 2 appears in samples incubated with inhibitors. As can be observed, the LV extracts not only inhibited the activity of α-amylase but also changed the profile of starch degradation products. 

The inhibition of α-amylase was calculated based on the peak area of maltose and the obtained results are presented as α-amylase activity [% control] in Figure 4C. Additionally, the results of the multiple comparison of samples using one-way ANOVA with Tukey’s test are attached as Supplementary Materials (Table S9). The strongest inhibitory effect from tested extracts showed post-seasonal fruits (FR_V), followed by flowers (FL_VI), leaves collected in July (L_VII) and ripe fruits (FR_IX) with a value *p* < 0.0001. The rest of the tested plant extracts showed weaker, but still statistically significant, inhibition of this enzyme (*p* < 0.001). The lowest yet still significant (*p* < 0.01) inhibitory effect was obtained for June leaves (L_VI). To the best of our knowledge, the ability of different morphological parts of LV extracts to inhibit the activity of α-amylase has not been tested so far. The only published data that we were able to find were results obtained for extracts of LV fruits that showed an inhibitory effect towards this enzyme [15]. 

### 2.6. Cyclooxygenase-2 (COX-2) Inhibitory Activity of L. vulgare Extracts

Cyclooxygenase (COX), also referred to as prostaglandin-endoperoxide synthase, functions as an enzyme responsible for creating essential biological agents known as prostanoids, which encompass prostaglandins, prostacyclin and thromboxane. COX plays a central role in the synthesis process of prostanoids from arachidonic acid. This enzyme has two recognized forms: COX-1 and COX-2. The form COX-1 is consistently present in numerous tissues and primarily prevails in the stomach’s mucous lining and the kidney. COX-2 remains inactive in most cells during regular circumstances, but its levels rise during instances of inflammation. As a consequence, COX-2 inhibitors are believed to be good anti-inflammatory agents. 

**Figure 5:**
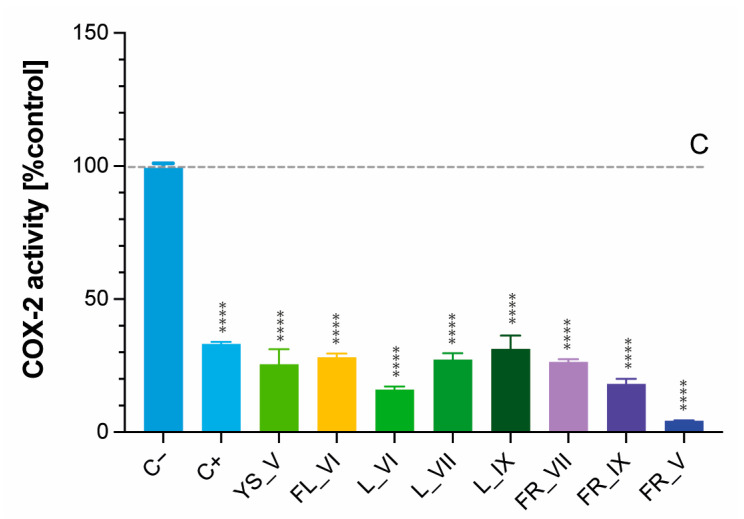
Anti-inflammatory activity of tested *Ligustrum vulgare* extracts determined as their ability to inhibit COX-2 activity. Results represent means ± SD. The abbreviations used: C− refer to the negative control, C+ refer to the positive control with inhibitor; FL, YS, L and FR refer to the morphological part of *Ligustrum vulgare* and mean, respectively: flowers, young shoots, leaves and fruits, while the numbers IV-IX indicate the month of their harvest. Significantly different values determined by using one-way analysis of variance (ANOVA) with Dunnet’s post-test are marked as **** *p* < 0.0001.

The obtained results showed that all tested *Ligustrum vulgare* extracts were good inhibitors of COX-2 activity, which proved their strong anti-inflammatory potential (Figure 5). Statistical analyses of the obtained results using one-way ANOVA with Tukey’s multiple comparison test are attached as Supplementary Materials (Table S10). All tested samples significantly inhibited COX-2 compared to the control (*p* < 0.0001). However, the young shoots, flowers, July and September leaves and unripe fruit (FR_VII) extracts exhibited no significant inhibition compared to the positive control (*p* > 0.05; Table S10). The strongest anti-inflammatory activity among all tested samples was observed in the case of post-seasonal fruits (FR_V) (*p* < 0.0001). Despite the fact that *Ligustrum vulgare* is well known in folk medicine as a remedy for inflammation, the only published data on the anti-inflammatory activity of this plant were for leaf extracts, and the obtained results were in line with our findings [41]. However, the heatmap obtained from UHPLC/HRMS analysis (Figure 2) indicates the high content of nuzhenide and its derivatives in ripe and post-seasonal fruits of *L*. *vulgare*. These compounds are known as one of the most abundant constituents in *Ligusturm lucidum* fruits with reported anti-inflammatory activity [8,18].

### 2.7. Antiproliferative Activity of L. vulgare Extracts

**Figure 6:**
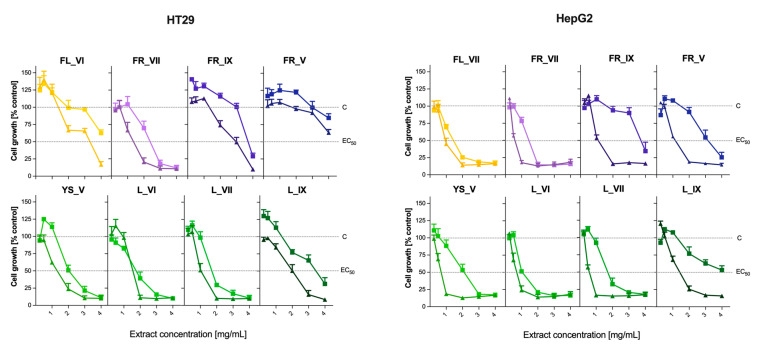
Growth inhibition of human colorectal cancer cells (HT29) and human liver cancer cells (HepG2) treated with *Ligustrum vulgare* extracts for 6 h (squares) and 24 h (triangles). The cytotoxicity was presented as a mg of dry mass of plant per mL of medium that inhibits the growth of cell line. The results represent means ± SD of three independent determinations. The abbreviations used: FL, YS, L and FR refer to the morphological part of *Ligustrum vulgare* and mean, respectively: flowers, young shoots, leaves and fruits, while the numbers IV-IX indicate the month of their harvest.

The antiproliferative effect of the tested *L. vulgare* extracts on human colorectal (HT29) and human liver (HepG2) cancer cell lines was determined using a standard MTT test. The antiproliferative effect was expressed as the growth inhibition of cells treated with LV extracts relative to a control treated with ethanol at a final concentration of 2.8% (treated as 100% cell growth). The obtained results are shown in Figure 6, while the EC50 values are presented in the Supplementary Materials in Table S11.

The cytotoxic effect of all tested extracts towards both cell lines increased with the concentration of the extract. However, in the case of the HT29 cell line, it was observed that in the case of a lower range of extract concentrations and a shorter (6 h) incubation time, there was a temporary increase in the cell growth compared to the control for some extracts (FL_VI, FR_IX, FR_V, YS_V, L_IX). This may indicate that some compounds present in these extracts induced the division of intestinal cells. This phenomenon has not been observed in the case of liver cells. The antiproliferative activity of *L. vulgare* extracts depended on the time of exposure, which was particularly observed in the HepG2 cell line treated with extracts of ripe and post-seasonal fruits (FR_IX, FR_V), young shoots and September leaves. HT29 cells appear to be less sensitive to post-seasonal fruit extract (FR_V) as it was the only one that did not achieve an EC50 value within the concentration range tested. The lowest EC50 values for both HT29 and HepG2 cells were obtained from June leaf (L_VI) extracts at concentrations of 1.0 mg d.w./mL (24 h) and 0.8 mg d.w./mL, respectively.

Ripe and post-seasonal fruits (FR_IX, FR_V) and leaves from September (L_IX) showed the lowest cytotoxic effect in relation to both tested cell lines. In addition, the HepG2 line was more sensitive to LV flower extract than the HT29 line. The strongest cytotoxic effect was shown primarily by young parts of LV (YS_V, L_VI, L_VII, FR_VII), where the survival of HepG2 and HT29 cells drastically decreased at the concentration of extracts of 1–1.5 mg/mL. Comparing the obtained results with heat maps (Figure 2), it can be concluded that the cytotoxic effect could be caused by quinic acid derivatives (especially cinnamic acid), whose concentration decreased at various stages of leaf maturity, and tyrosol derivatives, whose content was negligible in the case of ripe fruits and highest in leaf extracts. Our results correspond with the previously published data on the antiproliferative effects of LV extracts. Ćurčić et al. [42] examined the growth inhibitory effects of methanolic leaf and fruit extracts of *L. vulgare* on HCT-116 cells over different time periods. Their results show that the antiproliferative effects of LV extracts increase with the extension of exposure time. Zarić et al. [43] showed that LV leaves and fruit extracts exhibited a moderate cytotoxic effect on three types of leukemia cells. The *Ligustrum vulgare* leaf extract was the most effective on MOLT-4 cells, while the fruit extract was most effective on JVM-13 cells; both extracts were equally effective on CLL cells. In addition, none of the tested extracts was toxic to healthy mononuclear cells. Both extracts acted by inducing apoptosis of leukemic cells.

## 3. Materials and Methods

### 3.1. Chemicals and Reagents

The following chemicals and reagents were purchased from Sigma-Aldrich (Taufkirchen, Germany): 1-diphenyl-2-picrylhydrazyl (DPPH), 2,2-azinobis-(ethyl-2,3-dihydrobenzothiazoline-6-sulfonic acid) diammonium salt (ABTS), sodium thiosulfate, thiazolyl blue tetrazolium bromide (MTT), phosphate-buffered saline (PBS), dimethyl sulfoxide (DMSO), oleuropein (purity ≥ 98.0%, CAS-No: 32619-42-4), echinacoside (purity ≥ 98.0%, CAS-No: 82854-37-3), rutin (≥94.0%, CAS-No: 207671-50-9), 2-propanol, diphenylamine, aniline, McCoys’ 5A medium (modified, with sodium bicarbonate, without L-glutamine, liquid, sterile-filtered, suitable for cell culture), fetal bovine serum (FBS, non-USA origin, sterile-filtered, suitable for cell culture), penicillin–streptomycin (solution stabilized, with 10,000 units penicillin and 10 mg streptomycin/mL, 0.1 μm filtered, BioReagent, suitable for cell culture), formic acid, α-amylase from porcine pancreas (Type VI, 5 U/mg) and acarbose. Acetonitrile was from Merck (Darmstadt, Germany). Ethanol, methanol, sulfuric acid, phosphate acid, boric acid, 1-butanol, starch soluble and sodium persulfate were from POCH (Gliwice, Poland). Water was purified with a QPLUS185 system from Millipore (Burlington, MA, USA). 

### 3.2. Plant Material

The various morphological parts (young shoots, flowers, leaves and fruits) of common privet (*Ligustrum vulgare* L.) were collected at different development stages in Gdańsk, Poland. Botanical identification was made by Igor Kosiński. The voucher specimen was deposited in the Herbarium of the Medical University of Gdańsk, Poland (GDMA Herbarium No. 4638). The plant samples were frozen, lyophilized (Alpha 2–4 Christ LDC, Osterode am Harz, Germany), ground and kept at −20 °C until extract preparation. The sample abbreviations used in all analytical and biological assays are presented in Table 3.

### 3.3. Plant Extract Preparation

To simplify and reduce the number of samples in biological tests, the method of extraction was inspired by Liu et al. [44]. For the preparation of extracts, 100 mg of lyophilized plant samples of *Ligustrum vulgare* were suspended in 1 mL of 70% (*v*/*v*) ethanol. The resulting suspensions were mixed and sonicated for 15 min. After this time, the samples were centrifuged (13,200 rpm/15 min) in a Heraeus Megafuge 16R centrifuge (ThermoFisher Scientific, Dreieich, Germany). The resulting supernatant was collected into new tubes and centrifuged again under the same conditions. The purified extracts were transferred to new tubes and described as shown in Table 3. The extraction yield was 44%. The extracts were then used in further analysis.

### 3.4. Cell Culture

Human colon adenocarcinoma (HT29) cells and human hepatocellular carcinoma (HepG2) cells, obtained from ATCC, were cultured in McCoy’s medium and Dulbecco’s Modified Eagle Medium (DMEM), respectively. Culture media were supplemented with L-glutamine (2 mol/L), sodium pyruvate (200 g/L), fetal bovine serum (100 mL/L) and antibiotics (100 U/mL penicillin and 100 g/L streptomycin). The cells were maintained in a Smart cell incubator (Heal Force) under conditions of 37 °C and a humidified atmosphere containing 5% CO_2_, following the previously established protocol [45]. Routine checks for mycoplasma contamination in the cultured cells were conducted using the Universal Mycoplasma Detection Kit from ATCC (Manassas, VA, USA).

### 3.5. Metabolomic Analysis with LC-Q-Orbitrap HRMS

The crude extracts from different samples of *L. vulgare* were diluted 10 times with ethanol (70%) and analyzed using the UltiMate 3000 UHPLC system by Thermo Scientific Dionex. This UHPLC system was composed of a quaternary pump, a well plate autosampler, a column compartment equipped with a Luna Omega Polar C18, a 100 A° column (150 × 2.1 mm, 1.6 µm, Phenomenex) and a DAD detector. It was coupled with a high-resolution Thermo Q-ExactiveTM Focus quadrupole-Orbitrap mass spectrometer, manufactured by Thermo (Bremen, Germany). The entire chromatographic system was managed using Chromeleon 7.2.8 software from Thermo Fisher Scientific (Waltham, MA, USA). For the elution process, the mobile phases utilized were as follows: A—water acidified with formic acid (0.1%) and B—acetonitrile acidified with formic acid (0.1%). A constant flow rate of 0.3 mL/min was maintained for all separation procedures. The gradient commencing with 15% B, escalated to 30% B within 25 min, reached 100% B at the 27 min mark and was sustained at this level until 35 min. To condition the column, the initial mobile phase was run for a duration of 7 min. The injection volume was 5 μL. The analytes were ionized in negative ion mode through HESI (heated electrospray ionization). The flow rates for sheath gas, auxiliary gas and sweep gas were set at 35 bar, 15 bar and 3 bar, respectively. The spray voltage was maintained at 2.5 kV, and the S-lens RF level was set to 50. The capillary temperature and heater temperature were held at 350 °C and 300 °C, respectively. For the full MS scan, the mass range spanned from 120 to 1200 *m*/*z*, with a resolution of 70,000 FWHM (full width at half maximum). The AGC (automatic gain control) target was set at 2 × 10^5^, and the maximum injection time was established as 100 ms. When it came to MS2 parameters, the settings were as follows: a resolution of 17,500 FWHM, an isolation window of 3 *m*/*z*, a collision energy of 30 eV, an AGC target of 1 × 10^6^ and a maximum injection time of 100 ms. Subsequent data processing was carried out using Compound Discoverer 3.3 software and Freestyle 1.3 software.

### 3.6. Quantitative Analysis of Selected Phytochemicals by HPLC-DAD

To prepare the oleuropein, echinacoside and rutin standard solutions, 1 mg of the respective compounds were dissolved in 1 mL of ethanol. The calibration curve was generated by the integration of the areas of absorption peaks (270 nm for oleuropein and rutin and 325 nm for echinacoside) determined during HPLC-DAD analysis of serial dilutions of authentic standards. The limit of detection (LOD) was defined as the absolute amount of analyte that maintained a signal-to-noise ratio (peak height) of 3:1, while the limit of quantification (LOQ) was defined as the absolute amount of analyte that produced a signal-to-noise ratio of 10:1. Noise was the magnitude of background response, which was determined by analyzing blank samples (70% ethanol). A linear regression method was used to determine the regression coefficient (r2) and the linear equation. The chromatographic system, column and conditions of separation were the same as in the case of LC-Q-Orbitrap HRMS (Section 3.5).

### 3.7. Determination of Antioxidant Activity

The assessment of antioxidant activity using colorimetric methods employing ABTS and DPPH radicals was performed as outlined in a previous study [39], with slight adjustments. In brief, initial solutions of radicals were diluted in methanol until the absorbance reached 0.70 ± 0.02 at λ = 734 nm for ABTS radicals and 1.00 ± 0.02 at 515 nm for DPPH radicals. All reactions were conducted in 48-well plates. Before the analyses, a series of dilutions of crude LV extracts in the range of 1-5 mg d.w./mL were prepared using 70% ethanol. The mixture of 1 mL ABTS solution and 10 μL diluted plant extract was assessed for absorbance at 734 nm after 10 min, while the mixture of 1 mL DPPH solution and 30 μL diluted plant extract was measured at 515 nm after 10 min. Absorbance readings were carried out using a BioTek Synergy HT Microplate Reader spectrophotometer (Santa Clara, CA, USA). The results of antioxidant activity, determined through spectrophotometric tests, were expressed as stoichiometry values (n10), following the approach described by Kusznierewicz et al. [39]. In the case of ABTS and DPPH assays, this parameter was identified as a regression coefficient, representing the tangent of the line describing the relationship between concentrations of a radical scavenger and concentrations of the tested antioxidant in the mixture after a 10 min reaction (n10). The concentration of radicals scavenged by the tested antioxidants in the reaction media was calculated using the Beer–Lambert–Bouguer Law (Beer’s Law).

### 3.8. Determination of α-Amylase Inhibitory Activity 

To determine the antidiabetic activity of the tested LV extracts, a novel screening method for detecting α-amylase inhibitors in plant samples was used. This method is based on the determination of individual products of the enzymatic degradation of starch separated using high-performance thin-layer chromatography (HPTLC). The combination of chromatographic separation with densitometric detection of analytes determines the activity of α-amylase inhibitors. The concentration of individual components added to the reaction mixture was as follows: starch, 5 mg/mL dissolved in hot water, acarbose, 1 mg/mL dissolved in water, and amylase, 1 mg/mL (~5 U/mL) dissolved in PBS buffer. In this study, four types of reaction mixtures were prepared: positive control (400 μL starch + 200 μL amylase + 200 μL PBS), control with an inhibitor (400 μL starch + 200 μL amylase + 200 μL acarbose), plant samples (400 μL starch + 200 μL amylase + 200 μL plant extract) and sample background (600 μL PBS + 200 μL plant extract). The final concentration of the extracts that were used to test the ability to inhibit amylase was 25 mg d.w. of plant material per 1 mL of the reaction solution. In the first stage, all the ingredients, except for amylase, were mixed and incubated at 37 °C for 5 min. Then, 200 μL of α-amylase was added to the control and research samples. All mixtures were incubated for 30 min at 37 °C and then heated to 100 °C for 5 min to stop the enzymatic reaction.

The profiling of the products of the enzymatic reaction was processed using a CAMAG HPTLC system (Muttzenz, Switzerland). All post-reaction mixtures and background samples (2 μL) were applied as 6 mm bands onto glass silica gel HPTLC plates (20 × 10 cm, F254, Mereck, Darmstadt, Germany) by an automatic ATS4-TLC sampler. The chromatogram was developed in a previously saturated ADC2-TLC chamber using the mixture of 1-butanol, 2-propanol and boric acid (5 mg/mL), 30:50:10 (*v*/*v*/*v*), as a mobile phase. The conditions of development were as follows: tank saturation time 60 min, plate preconditioning time 5 min, relative humidity 33%, plate drying time 5 min and migration distance 65 mm. After drying, the developed HPTLC plates were photographed under white light at 254 nm and 366 nm (TLC visualizer 2). To visualize the products of enzymatic starch decomposition, the derivatization reagent was prepared by dissolving 2 g of diphenylamine and 2 mL of aniline in 80 mL of methanol, then adding 10 mL of phosphoric acid (85% *v*/*v*) and filling with methanol to 100 mL. The TLC plate was sprayed with 2 mL of the reagent by an automatic spraying TLC Derivatizer and then heated for 5 min at 100 °C. After cooling, the plates were photographed under transmission white light. The spectrodensitometric analysis was performed in the absorption mode at 380 nm by a TLC Scanner 4. Based on the obtained profiles and densitograms, the ability of the tested plant extracts to inhibit α-amylase was assessed.

### 3.9. Determination of Cyclooxygenase-2 Inhibitory Activity

The ability of the tested plant extracts to inhibit COX-2 activity was determined using a commercially available COX-2 Inhibitor screening kit (Sigma Aldrich, Taufkirchen, Germany), strictly following the manufacturer’s recommendations. The results were presented as COX-2 inhibition [% control]. The final concentration of the extracts that were used to test the ability to inhibit cyclooxygenase-2 (COX-2) was 1 mg d.w. of plant material per 1 mL of reaction solution.

### 3.10. Determination of Cytotoxicity by MTT Test

The MTT assay was conducted to evaluate the potential of the examined plant extracts to inhibit the proliferation of HT29 and HepG2 cells, following the previously outlined methodology [45]. To elaborate, HT29 or HepG2 cells were seeded at a density of 10^4^ cells per well in 96-well tissue culture plates containing 0.15 mL of appropriate medium. Following a 24 h incubation period at 37 °C for cell settling, different concentrations of the tested extracts were introduced into the cell culture medium, and the cells were incubated for either 6 or 24 h. In the case of shorter exposure, the well contents were replaced with 0.2 mL of fresh medium, and the cells were further incubated at 37 °C to complete a total 24 h incubation period. After the 24 h incubation, a solution of MTT (4 g/L) was added (0.05 mL per well), and the multiwell plate was subjected to an additional 4 h incubation at 37 °C. Following this, the medium was meticulously aspirated from the wells, and the resulting formazan crystals generated by metabolically active cells were dissolved in 0.05 mL of DMSO. The absorbance of the resulting solutions was measured at 540 nm using a BioTek Synergy HT Microplate Reader spectrophotometer (Santa Clara, CA, USA). Each treatment was independently replicated three times. The cytotoxicity was expressed as a percentage of cell growth for cells exposed to the tested extracts relative to control cells treated solely with the appropriate volume of solvent; the growth of the latter was considered 100%.

### 3.11. Statistical Analysis

Unless stated otherwise, the presented values represent the means ± standard deviation (SD) derived from three distinct measurements. Statistical significance was evaluated using either one-way ANOVA with the Tukey–Kramer test or one-way ANOVA with Dunnett’s test. All statistical computations were carried out using Prism 10.1.1. software package provided by GraphPad Software, Inc. (Boston, MA, USA). The threshold for statistical significance was defined as *p* ≤ 0.05.

## 4. Conclusions

In conclusion, our investigation on *Ligustrum vulgare*, commonly known as common privet, has shed some light on its potential therapeutic properties. The traditional uses attributed to this species in folk medicine find support in our comprehensive study.

Our study encompassed various morphological parts of *Ligustrum vulgare*, including young shoots, leaves, flowers and fruits harvested at different developmental stages, in its metabolomics analyses. This approach revealed that extracts from different parts of *Ligustrum vulgare* are abundant sources of diverse bioactive phytochemicals such as phenylethanoids, triterpenes, flavonoids, organic acids, lignans and the characteristic iridoids associated with this plant family.

Significantly, our findings underscore the remarkable antioxidant potential exhibited by *Ligustrum vulgare* extracts, as well as their notable anti-inflammatory, antidiabetic and antiproliferative activities. Bioactive compounds, present in varying concentrations across different morphological parts, contribute to the multifaceted medicinal properties attributed to this plant.

Of particular note is the pioneering nature of our study, providing the first comprehensive analysis of *Ligustrum vulgare* across its distinct morphological components. This in-depth exploration enhances our understanding of the plant’s potential therapeutic applications and lays the groundwork for future investigations into harnessing its bioactive compounds for medicinal purposes. As *Ligustrum vulgare* continues to reveal its pharmacological potential, our study contributes valuable insights into the diverse array of health-promoting compounds present in this traditionally esteemed plant.

## Figures and Tables

**Table 1:** Retention time (Rt, min), proposed formula, theoretical mass of the parent ion (Da), experimental mass of the parent ion (Da), accuracy (Δm, ppm) and the most intensive mass fragments (Da) of peaks tentatively identified in *Ligustrum vulgare* extracts with the use of LC-Q-Orbitrap HRMS in negative ion mode.

No	Rt [min]	Compound	Formula	Theoretical [M − H]^−^	Experimental [M − H]^−^	Δm [ppm]	MS/MS	Ref.
Organic acids and derivatives								
1	1.58	D-(-)-Quinic acid	C_7_H_12_O_6_	191.05557	191.05513	2.28	85.028; 93.033; 59.01238; 71.012; 109.028	[18,19]
2	1.74	Citric acid	C_6_H_8_O_7_	191.01918	191.01891	1.44	87.007; 85.028; 111.007	[19]
6	2.91	Caffeoylquinic acid 1	C_16_H_18_O_9_	353.08726	353.08771	−1.27	191.055; 135.044; 179.034; 136.047; 161.023; 180.037; 85.028; 173.044; 111.044	[19]
12	3.66	Syringin *	C_17_H_24_O_9_	417.13969	417.14023	−1.29	209.081; 194.058; 176.047; 328.599; 268.111; 318.322; 259.172; 356.034; 117.326	[20]
13	3.75	*p*-Coumaroylhexaric acid	C_15_H_16_O_10_	355.06653	355.06705	−1.47	85.028; 57.033; 209.030; 191.019; 59.012; 129.018; 133.013; 86.031; 111.007	[21]
15	4.11	*p*-Coumaroyl acid hexoside 1	C_15_H_18_O_8_	325.09235	325.09287	−1.60	119.049; 163.039; 120.052; 91.513; 174.794; 236.837; 183.034; 232.073; 167.113	[22]
16	4.48	Caffeoylquinic acid 2	C_16_H_18_O_9_	353.08726	353.08783	−1.61	135.044; 173.044; 191.055; 179.034; 93.033; 137.023; 136.047; 174.048; 85.028	[19]
17	4.50	Vanilin	C_8_H_8_O_3_	151.03952	151.03893	3.94	123.044; 93.033; 108.020; 121.028; 95.049; 105.033; 67.017; 77.038; 137.023	[19,23]
18	4.68	*p*-Coumaroyl acid hexoside 2	C_15_H_18_O_8_	325.09235	325.09293	−1.79	145.028; 117.033; 146.032; 59.012; 119.049; 89.023; 163.039; 101.023; 161.060	[22]
22	6.17	Esculetin	C_9_H_6_O_4_	177.01879	177.01837	2.34	89.038; 105.033; 133.028; 93.033; 81.033; 95.049; 121.028; 149.023; 177.018	[19,23]
24	6.70	*p*-Cumaroylquinic acid	C_16_H_18_O_8_	337.09235	337.09283	−1.45	173.044; 93.033; 119.049; 163.039; 111.044; 137.023; 174.048; 155.034; 67.017	[22]
26	7.58	Feruloylquinic acid	C_17_H_20_O_9_	367.10291	367.10327	−0.98	173.044; 93.033; 134.036; 193.050; 111.044; 174.048; 137.023; 155.034; 67.017	[24]
30	10.26	*p*-Coumaric acid	C_9_H_8_O_3_	163.03952	163.03900	3.18	119.049; 93.033; 120.052; 117.033; 91.054; 65.038; 94.036; 104.0253; 103.124	[22]
51	14.35	Cinnamoylquinic acid	C_16_H_18_O_7_	321.09743	321.09778	−1.08	173.044; 93.033; 147.044; 111.044; 137.023; 174.048; 71.012; 155.034; 59.012	-
Phenylethanoids								
3	2.26	Hydroxytyrosol glucoside	C_14_H_20_O_8_	315.10800	315.10852	−1.67	123.044; 153.054; 124.047; 154.058; 59.012; 71.012; 135.044; 89.023; 101.023; 108.020	[22]
4	2.38	Bioside	C_20_H_30_O_12_	461.16591	461.16644	−1.17	113.023; 135.044; 71.012; 89.023; 461.166; 59.012; 101.023; 315.108; 85.028; 161.044	[25]
7	2.92	Salidroside (tyrosol glucoside)	C_14_H_20_O_7_	299.11308	299.11346	−1.29	59.012; 71.012; 119.049; 89.023; 101.023; 85.028; 113.023; 95.012; 58.005; 137.059	[18]
8	2.94	Osmanthuside H	C_19_H_28_O_11_	431.15534	431.15558	−0.55	89.023; 59.012; 101.023; 71.012; 119.034; 119.049; 149.044; 113.023; 131.034; 191.055	[18]
9	3.08	Hydroxytyrosol	C_8_H_10_O_3_	153.05517	153.05458	3.85	123.044; 122.036; 95.049; 93.033; 108.020; 81.033; 67.017; 121.028; 95.012; 124.047	[18]
25	7.50	Echinacoside 1	C_35_H_46_O_20_	785.25043	785.25067	−0.31	785.251; 623.219; 161.023; 179.034; 786.249; 91.514; 623.141; 434.588; 463.301; 58.550	ST
29	10.26	Echinacoside 2	C_35_H_46_O_20_	785.25043	785.25110	−0.86	785.250; 786.254; 623.219; 161.023; 624.221; 162.027; 366.595; 384.669; 106.970; 363.523	[18]
40	12.31	Hydroxytyrosol acetate	C_10_H_12_O_4_	195.06574	195.06544	1.49	59.012; 60.016; 166.294; 142.985; 177.482; 210.096; 99.362; 159.217; 73.254; 58.937	[22]
44	12.92	Verbascoside 1	C_29_H_36_O_15_	623.19760	623.19794	−0.54	161.023; 623.198; 461.166; 135.044; 179.034; 113.023; 315.111; 133.027; 71.012; 305.068	[18,22]
56	14.85	Verbascoside 2	C_29_H_36_O_15_	623.19760	623.19812	−0.83	623.198; 161.023; 624.201; 461.166; 462.170; 162.027; 135.044; 113.023; 251.056; 179.034	[18,22]
59	15.22	Verbascoside 3	C_29_H_36_O_15_	623.19760	623.19788	−0.44	161.023; 623.198; 461.166; 624.202; 162.027; 462.170; 135.044; 179.034; 113.023; 315.108	[18,22]
65	16.44	Isosyringalide rhamnoside 1	C_29_H_36_O_14_	607.20269	607.20331	−1.03	145.028; 461.166; 607.203; 163.039; 462.170; 146.0317; 608.206; 113.023; 135.044; 315.109	[7]
68	16.79	Syringalide A	C_23_H_26_O_10_	461.14478	461.14523	−0.99	161.023; 461.145; 162.0267; 462.149; 179.034; 135.044; 133.028; 89.023; 101.023; 221.045	[26]
71	17.57	Isosyringalide rhamnoside 2	C_29_H_36_O_14_	607.20269	607.20325	−0.93	145.028; 461.166; 607.203; 462.170; 163.039; 608.208; 146.032; 113.023; 135.044; 153.055	[7]
Flavonoids								
11	3.65	Taxifolin-3-*O-*glucoside	C_21_H_22_O_12_	465.10331	465.10364	−0.72	285.040; 125.023; 275.056; 178.998; 177.018; 181.013; 153.018; 151.002; 303.051	[23]
14	4.00	Cyanidin-3-*O*-rutinoside	C_27_H_30_O_15_	593.15065	593.15125	−1.00	285.040; 284.032; 593.151; 594.155; 286.044; 299.056; 125.023; 149.044; 89.023	[27]
32	10.69	Luteolin-4’,7-*O*-diglucoside	C_27_H_30_O_16_	609.14557	609.14600	−0.71	284.032; 285.040; 609.146; 610.149; 286.043; 429.081; 178.998; 283.024; 257.044	[22]
36	11.71	Eriodyctiol glucoside	C_21_H_22_O_11_	449.10839	449.10898	−1.31	151.002; 287.056; 135.044; 288.060; 175.003; 152.006; 125.023; 193.014; 68.994	[23]
38	12.14	Quercetin-3-*O*-rutinoside	C_27_H_30_O_16_	609.14557	609.14575	−0.31	609.146; 301.035; 300.027; 610.149; 302.039; 178.998; 343.046; 302.006; 179.195	ST
39	12.28	Luteolin-7-*O*-rutinoside	C_27_H_30_O_15_	593.15065	593.15106	−0.69	285.040; 593.151; 594.154; 286.044; 273.076; 274.079; 307.082; 361.094; 327.052	[22]
46	13.40	Kaemferol-7-*O*-glucoside	C_21_H_20_O_11_	447.09274	447.09311	−0.83	285.040; 284.032; 447.093; 448.097; 327.051; 284.000; 269.046; 297.039	[18]
47	13.43	Kaemferol-3-*O*-rutinoside	C_27_H_30_O_15_	593.15065	593.15094	−0.49	593.151; 594.154; 285.040; 284.032; 447.093; 286.045; 448.097; 91.513; 594.254; 327.049	[18]
53	14.44	Ligustroflavone	C_33_H_40_O_19_	723.21365	723.21362	0.03	723.214; 724.218; 269.045; 270.049; 577.159; 559.147; 268.036; 428.833; 159.412; 103.969	[7]
58	15.22	Luteolin-7-*O*-rhamnoside	C_27_H_30_O_15_	593.15065	593.15094	−0.49	285.040; 593.151; 284.032; 594.154; 286.044; 327.051; 151.002; 257.047; 444.174; 99.526	[22]
61	15.65	Apigenin-7-*O*-rutinoside 1	C_27_H_30_O_14_	577.15574	577.15607	−0.58	269.045; 270.048; 577.156; 311.056; 65.002; 578.155; 67.388; 63.022; 64.999; 91.507	[18]
64	16.12	Naringenin hexoside	C_21_H_22_O_10_	433.11348	433.11392	−1.03	271.061; 151.002; 272.065; 119.049; 112.984; 93.033; 68.994; 177.018; 152.006; 175.002	[23]
66	16.68	Apigenin-7-*O*-rutinoside 2	C_27_H_30_O_14_	577.15574	577.15607	−0.58	269.045; 577.156; 578.160; 270.049; 268.037; 413.088; 311.056; 431.100; 457.112; 101.023	[18]
70	17.22	Apigenin-7-*O*-glucoside	C_21_H_20_O_10_	431.09783	431.09821	−0.88	268.038; 269.044; 431.098; 432.101; 270.049; 311.056; 283.060; 281.046; 341.068; 151.002	[18,22]
73	18.03	Chrysoeriol-7-*O*-glucoside	C_22_H_22_O_11_	461.10839	461.10870	−0.68	446.085; 461.109; 283.024; 298.048; 299.054; 447.089; 462.112; 284.030; 313.035; 297.040	[19]
91	24.17	Eriodictyol	C_15_O_12_O_6_	287.05557	287.05603	−1.62	135.044; 151.002; 107.012; 65.002; 136.047; 83.012; 63.022; 109.028; 152.006; 108.016	[23]
98	26.74	Luteolin	C_15_H_10_O_6_	285.03992	285.04041	−1.72	133.028; 285.040; 151.002; 175.039; 107.012; 149.023; 199.039; 286.044; 217.050; 134.031	[18,23]
106	29.31	Naringenin	C_15_H_12_O_15_	271.06065	271.06134	−2.55	119.049; 107.012; 151.003; 65.002; 83.012; 63.023; 187.039; 120.052; 93.033; 161.060	[20,23]
107	29.40	Apigenin	C_15_H_10_O_5_	269.04500	269.04541	−1.52	117.033; 151.002; 149.023; 269.045; 107.012; 65.002; 225.055; 118.036; 121.028; 159.044	[18,22]
Iridoids								
10	3.39	Oleoside	C_16_H_22_O_16_	389.10839	389.10898	−1.51	69.033; 59.012; 121.064; 89.023; 71.012; 95.049; 101.023; 113.023; 165.054; 119.034	[18]
20	5.09	Genipin 1-gentiobioside	C_23_H_34_O_15_	549.18195	549.18243	−0.88	101.023; 163.060; 205.071; 59.012; 103.039; 89.023; 143.034; 73.028; 119.034; 71.012	-
21	5.16	Oleoside 11-methyl ester 1	C_17_H_24_O_11_	403.12404	403.12439	−0.87	59.012; 89.023; 71.012; 101.023; 113.023; 69.033; 119.033; 121.028; 85.028; 127.038	[18]
23	6.29	Oleoside 11-methyl ester 2	C_17_H_24_O_11_	403.12404	403.12448	−1.09	59.012; 89.023; 71.012; 101.023; 113.023; 119.034; 197.081; 85.028; 68.997; 165.055	[18]
27	8.79	Elenolic acid	C_11_H_14_O_6_	241.07122	241.07141	−0.81	67.017; 68.997; 111.007; 95.049; 127.039; 139.002; 101.023; 121.028; 69.033; 123.043	[18]
28	10.01	Genipin	C_11_H_14_O_5_	225.07630	225.07649	−0.85	68.997; 101.023; 67.017; 127.039; 106.041; 123.044; 108.020; 70.000; 95.049; 125.023	-
31	10.29	7-Hydroxyoleuropein	C_25_H_32_O_14_	555.17139	555.17145	−0.11	151.039; 89.023; 223.060; 152.042; 101.023; 119.034; 59.012; 71.012; 149.023; 113.023	[18]
34	10.92	Neonuzhenide 1	C_31_H_42_O_18_	701.22930	701.22986	−0.80	315.108; 469.135; 437.148; 701.230; 316.111; 539.176; 470.138; 702.231; 357.119; 507.151	[18]
35	11.33	Oleuropeinic acid	C_25_H_30_O_15_	569.15065	569.15088	−0.40	151.039; 209.045; 331.082; 177.018; 123.044; 183.065; 89.023; 165.054; 195.029; 221.008	[18]
37	12.10	10-Hydroxyoleuropein 1	C_25_O_32_O_14_	555.17139	555.17163	−0.44	273.077; 89.023; 137.023; 119.033; 101.023; 111.044; 59.012; 307.082; 181.050; 275.056	[18]
41	12.55	Nuzhenide 1	C_31_H_42_O_17_	685.23438	685.23474	−0.53	453.140; 299.113; 223.061; 89.023; 101.023; 421.150; 119.034; 179.055; 59.012; 121.028	[18,22]
42	12.61	10-Hydroxyoleuropein 2	C_25_H_32_O_14_	555.17139	555.17163	−0.44	195.065; 273.077; 239.055; 89.023; 361.093; 307.082; 137.023; 387.093; 119.034; 101.023	[18,23]
43	12.84	Neonuzhenide 2	C_31_H_42_O_18_	701.22930	701.22961	−0.46	315.108; 316.112; 135.044; 59.013; 89.023; 119.033; 179.055; 101.022; 59.009; 153.054	[18]
45	13.13	10-Hydroxyoleuropein 3	C_25_H_32_O_14_	555.17139	555.17181	−0.77	195.065; 273.077; 239.056; 89.023; 361.093; 307.082; 387.094; 137.023; 119.034; 101.023	[18]
49	14.08	Nuzhenide 2	C_31_H_42_O_17_	685.23438	685.23499	−0.88	453.140; 421.151; 299.113; 101.023; 223.061; 454.144; 89.023; 523.182; 119.034; 422.155	[18]
50	14.22	Nuzhenide 3	C_31_H_42_O_17_	685.23438	685.23505	−0.97	453.140; 421.151; 299.114; 101.023; 223.060; 89.023; 454.143; 523.182; 119.034; 422.154	[18]
52	14.38	Oleuropein dihexoside	C_31_H_42_O_18_	701.22930	701.22961	−0.46	539.177; 377.124; 275.092; 307.082; 540.180; 469.135; 179.055; 378.127; 437.146; 223.0604	[22,23]
57	15.14	Neonuzhenide 3	C_31_H_42_O_18_	701.22930	701.22961	−0.46	315.108; 316.111; 179.055; 275.092; 307.082; 135.044; 377.124; 89.023; 701.229; 223.060	[18]
60	15.29	Nuzhenide 4	C_31_H_42_O_17_	685.23438	685.23431	0.10	299.113; 223.060; 89.023; 623.198; 119.034; 101.023; 179.055; 113.023; 300.117; 71.012	[18]
62	15.91	10-Hydroxyligstroside 1	C_25_H_32_O_13_	539.17647	539.17706	−1.10	291.087; 101.023; 275.092; 111.044; 89.023; 127.039; 377.124; 59.012; 292.091; 239.056	[25]
63	16.03	Comselogoside 1	C_25_H_28_O_13_	535.14517	535.14581	−1.20	265.071; 205.050; 163.039; 235.061; 183.065; 145.028; 59.012; 121.064; 69.033; 177.055	[22]
67	16.73	Caffeoyl-6’-secologanoside	C_25_H_28_O_14_	551.14009	551.14056	−0.87	161.023; 281.066; 507.151; 389.109; 162.027; 179.034; 345.119; 251.056; 59.012; 323.077	[22]
69	16.90	10-Hydroxyligstroside 2	C_25_H_32_O_13_	539.17647	539.17706	−1.10	101.023; 89.023; 291.087; 221.045; 275.092; 153.055; 59.012; 119.034; 211.061; 71.012	[25]
72	17.91	Oleacein 1	C_17_H_20_O_6_	319.11817	319.11874	−1.81	69.033; 59.012; 95.049; 70.036; 139.075; 123.044; 139.039; 113.023; 96.052; 67.017	[22]
74	18.74	Oleacein 2	C_17_H_20_O_6_	319.11817	319.11853	−1.14	69.033; 59.012; 95.049; 70.036; 139.075; 139.039; 123.044; 96.052; 113.023; 67.017	[22]
75	19.03	Oleuropein 1	C_25_H_32_O_13_	539.17647	539.17676	−0.53	89.023; 275.093; 307.082; 95.049; 149.023; 59.012; 101.023; 139.039; 275.056; 119.034	ST
76	19.40	Oleacein 3	C_17_H_20_O_6_	319.11817	319.11874	−1.81	69.033; 59.012; 95.049; 70.036; 139.075; 139.039; 123.044; 96.052; 113.023; 107.049	[22]
77	19.73	Oleacein 4	C_17_H_20_O_6_	319.11817	319.11868	−1.62	69.033; 59.012; 95.049; 70.036; 139.075; 139.039; 113.023; 123.044; 96.052; 67.017	[22]
78	19.88	Oleacein 5	C_17_H_20_O_6_	319.11817	319.11874	−1.81	69.033; 59.012; 95.049; 107.049; 70.036; 137.059; 139.039; 121.028; 113.023; 139.075	[22]
79	20.06	G-13/Oleonuezhenide 1	C_48_H_64_O_27_	1071.35568	1071.35584	−0.14	1071.355; 771.235; 685.235; 1072.358; 523.182; 772.239; 403.124; 909.303; 686.238; 910.307	[18]
80	20.52	Oleuropein 2	C_25_H_32_O_13_	539.17647	539.17706	−1.10	89.023; 275.093; 149.023; 95.049; 59.012; 307.082; 101.023; 139.039; 119.034; 275.057	[18]
81	20.64	Comselogoside 2	C_25_H_28_O_13_	535.14517	535.14587	−1.32	145.028; 121.064; 163.039; 205.050; 59.0124; 265.071; 69.033; 146.032; 345.119; 165.055	[22]
82	20.72	Nuzhenide aglycone	C_25_H_32_O_12_	523.18156	523.18219	−1.21	101.023; 89.023; 119.034; 59.012; 121.028; 453.141; 71.012; 113.023; 119.048; 137.060	[18]
83	21.46	Oleoacteooside	C_46_H_58_O_25_	1009.31890	1009.31897	−0.07	1009.319; 1010.323; 847.278; 623.199; 848.278; 745.234; 665.209; 815.238; 161.023; 777.227	[25]
84	21.84	Lucidumoside C	C_27_H_36_O_14_	583.20269	583.20337	−1.17	151.039; 89.023; 223.061; 119.034; 101.023; 59.012; 179.055; 71.012; 152.042; 113.023	[18]
85	22.23	Oleuropein aglycone 1	C_19_H_22_O_8_	377.12365	377.12421	−1.49	95.049; 111.007; 139.002; 101.023; 139.039; 149.023; 127.039; 69.033; 68.997; 59.0124	[18]
86	22.37	Comselogoside 3	C_25_H_28_O_13_	535.14517	535.14569	−0.97	145.028; 121.064; 163.039; 265.071; 205.050; 69.033; 146.032; 59.012; 345.119; 165.054	[22]
87	22.55	Oleuropein aglycone 2	C_19_H_22_O_8_	377.12365	377.12421	−1.49	95.049; 111.007; 139.002; 139.039; 101.023; 149.023; 127.039; 69.033; 59.012; 68.996	[18]
88	22.83	10-Hydroxyligstroside aglycone 1	C_19_H_22_O_8_	377.12365	377.12402	−1.00	101.023; 127.039; 111.007; 68.997; 111.044; 139.002; 85.028; 171.029; 93.033; 137.059	[25]
89	22.96	Excelside B	C_31_H_42_O_17_	685.23438	685.23468	−0.44	291.087; 361.129; 259.097; 101.023; 113.023; 292.091; 223.061; 161.044; 362.132; 127.039	[25]
90	23.84	Ligustroside 1	C_25_H_32_O_12_	523.18156	523.18201	−0.86	291.087; 101.023; 259.097; 127.039; 292.091; 111.007; 69.033; 139.039; 89.023; 68.997	[18]
92	24.24	10-Hydroxyligstroside aglycone 2	C_19_H_22_O_8_	377.12365	377.12408	−1.17	101.023; 111.007; 127.039; 95.049; 139.002; 68.997; 111.044; 85.028; 171.029; 139.039	[25]
93	24.62	GL-3/Oleonezuhine 2	C_48_H_64_O_27_	1071.35568	1071.35547	0.20	685.235; 686.238; 523.182; 771.235; 1071.355; 1072.359; 403.124; 909.303; 772.239; 524.185	[18]
94	25.65	6′-*O*-*trans*-Cinnamoyl 8-epikingisidic acid	C_25_H_28_O_12_	519.15026	519.15070	−0.85	121.064; 147.044; 161.060; 59.012; 189.055; 69.033; 183.065; 165.055; 95.049; 122.068	[18]
95	25.66	Ligustroside 2	C_25_H_32_O_12_	523.18156	523.18201	−0.86	101.023; 291.087; 223.060; 89.023; 59.012; 361.129; 127.0387; 259.097; 71.012; 292.091	[18]
96	25.86	Oleuropein aglycone 3	C_19_H_22_O_8_	377.12365	377.12408	−1.17	95.049; 111.007; 139.039; 101.023; 139.002; 149.023; 127.039; 69.033; 68.997; 59.012	[18]
97	26.71	GL-3/Oleonezuhine 3	C_48_H_64_O_27_	1071.35568	1071.35559	0.08	1071.355; 685.235; 1072.358; 909.303; 523.182; 910.306; 686.238; 771.234; 403.124; 839.261	[18]
99	27.45	Ligustroside aglycone 1	C_19_H_22_O_7_	361.12873	361.12924	−1.42	101.023; 127.039; 111.007; 68.997; 69.033; 139.002; 171.029; 153.018; 137.059; 67.017	[22]
100	28.64	Oleuropein aglycone 4	C_19_H_22_O_8_	377.12365	377.12421	−1.49	95.049; 111.007; 139.002; 101.023; 139.039; 149.023; 127.039; 69.033; 59.012; 121.028	[22]
101	28.83	Jaspolyoside	C_42_H_54_O_23_	925.29777	925.29846	−0.75	539.177; 540.180; 377.124; 925.298; 926.301; 275.092; 307.082; 521.166; 378.127; 403.124	[19]
102	28.95	Oleuropein 3	C_25_H_32_O_13_	539.17647	539.17706	−1.10	89.023; 275.093; 307.082; 95.049; 149.023; 101.023; 59.012; 139.039; 275.057; 119.033	[18]
103	29.01	10-Hydroxyoleuropein 4	C_25_H_32_O_14_	555.17139	555.17169	−0.55	195.065; 273.077; 89.023; 239.055; 361.093; 307.082; 137.023; 387.093; 119.034; 101.023	[18]
104	29.04	6′-*O*-*cis*-Cinnamoyl-8-epikingisidic acid	C_25_H_28_O_12_	519.15026	519.15045	−0.38	147.044; 69.033; 121.064; 161.060; 59.012; 165.054; 189.055; 95.049; 121.028; 139.002	[18]
105	29.25	Oleuropein 4	C_25_H_32_O_13_	539.17647	539.17719	−1.33	89.023; 101.023; 275.093; 307.082; 95.049; 59.012; 149.023; 119.034; 139.039; 275.057	[22]
108	29.43	Ligustroside aglycone 2	C_19_H_22_O_7_	361.12873	361.12927	−1.50	101.023; 127.039; 111.007; 68.997; 69.033; 139.002; 171.029; 153.018; 67.017; 139.039	[22]
109	29.49	Oleuropein aglycone 5	C_19_H_22_O_8_	377.12365	377.12408	−1.17	95.049; 111.007; 139.002; 101.023; 139.039; 149.023; 127.039; 69.033; 68.997; 59.012	[18]
Lignans								
5	2.40	Acyclodihydroelenolic acid hexoside	C_17_H_28_O_11_	407.15534	407.15570	−0.89	59.012; 71.012; 151.075; 101.023; 89.023; 113.023; 85.028; 121.064; 73.028; 99.007	[19]
19	4.86	Cycloolivil glucoside	C_26_H_34_O_12_	537.19721	537.19757	−0.68	179.070; 195.065; 375.145; 191.070; 180.074; 376.148; 327.124; 345.134; 196.069; 360.121	[19]
33	10.87	Lariciresinol-4-*O*-glucoside	C_26_H_34_O_11_	521.20229	521.20221	0.15	329.139; 330.143; 349.150; 175.075; 485.203; 178.062; 169.086; 350.153; 71.012; 101.023	[25]
48	13.83	Pinoresinol-4-*O*-glucoside	C_26_H_32_O_11_	519.18664	519.18695	−0.60	151.039; 357.134; 358.138; 152.042; 342.110; 136.015; 175.075; 311.128; 71.012; 101.023	[25]
54	14.58	Syringaresinol-*O*-glucoside	C_28_H_36_O_13_	579.20777	579.20837	−1.04	417.155; 181.050; 418.159; 402.131; 182.053; 166.026; 403.134; 205.086; 371.150; 387.108	[28]
55	14.79	Medioresinol-4-*O-*glucopiranoside	C_27_H_34_O_12_	549.19721	549.19775	−1.00	387.145; 151.039; 181.050; 149.044; 89.023; 388.148; 101.023; 372.121; 131.034; 191.055	[25]
Triterpenes								
110	30.34	Tormentic acid	C_30_H_48_O_5_	487.34235	487.34271	−0.74	469.332; 487.343; 470.335; 488.346; 486.330; 423.325; 440.325; 467.317; 425.341; 424.328	[18]
111	31.36	Colosic acid	C_30_H_48_O_4_	471.34744	471.34763	−0.40	471.348; 472.351; 61.987; 91.507; 117.966; 222.480; 102.313; 294.133; 221.898; 326.910	[18]
112	31.51	3β-*O*-*cis*/*trans*-*p*-Coumaroylmaslinic acid	C_39_H_54_O_6_	617.38422	617.38446	−0.40	617.385; 618.388; 619.391; 145.028; 497.328; 119.049; 146.032; 645.004; 73.177; 105.122	[18]
113	32.57	Ursolic/oleanolic acid	C_30_H_48_O_3_	455.35252	455.35294	−0.91	455.353; 456.356; 97.360; 118.599; 50.633	[18]
114	33.65	Acetyloleanolic/ursolic acid	C_32_H_50_O_4_	497.36309	497.36328	−0.39	497.363; 498.367; 437.340; 429.303; 124.469; 91.513; 61.987; 323.325; 339.940; 111.674	[18]

* Pseudo-molecular ion detected as a formic acid adduct [M – H + FA]^−^; ST, identification confirmed based on an appropriate reference compound.

**Table 2:** Echinacoside, quercetin-3-*O*-rutinoside (rutin) and oleuropein content (mg/g d. w.) determined in *Ligustrum vulgare* extracts by UHPLC-DAD. The abbreviations: FL, YS, L and FR refer to the morphological part of *Ligustrum vulgare* and mean, respectively: flowers, young shoots, leaves and fruits, while the numbers IV-IX indicate the month of their harvest. Numbers followed by the same letters within one row are not significantly different according to one-way ANOVA with Tukey’s multiple comparison test at *p* ≤ 0.05.

No.	Compound	Content (mg/g d.w.)							
		YS_V	FL_VI	L_VI	L_VII	L_IX	FR_VII	FR_IX	FR_V
**25**	Echinacoside	17.13 ± 0.32 (a)	14.31 ± 0.21 (b)	14.12 ± 0.41 (b)	12.62 ± 0.37 (c)	9.51 ± 0.36 (d)	28.88 ± 0.21 (e)	7.44 ± 0.88 (f)	9.32 ± 0.17 (d)
**38**	Quercetin-3-*O*-rutinoside	9.54 ± 0.26 (a)	10.95 ± 0.42 (ab)	9.78 ± 0.32 (a)	11.29 ± 0.59 (b)	13.75 ± 0.96 (c)	4.92 ± 0.27 (de)	6.08 ± 0.58 (d)	3.89 ± 0.16 (e)
**75**	Oleuropein	26.05 ± 1.07 (a)	33.43 ± 2.48 (b)	18.85 ± 3.05 (c)	15.30 ± 0.83 (cd)	33.20 ± 1.83 (b)	10.29 ± 2.40 (d)	3.15 ± 0.52 (e)	0.67 ± 0.23 (e)

Echinacoside: linear range, 50–200 µg/mL; standard curve, y = 8122x − 236909; r^2^ = 0.990; LOD, 2.0 µg/mL; LOQ, 6.2 µg/mL. Rutin: linear range 30–300 µg/mL; standard curve, y = 28539x − 325274; r^2^ = 0.993; LOD, 0.5 µg/mL; LOQ, 2.0 µg/mL. Oleuropein: linear range, 10–300 µg/mL; standard curve, y = 3602x + 7065; r^2^ = 0.992; LOD, 4.2 µg/mL; LOQ, 14.0 µg/mL.

**Table 3:** Sample abbreviations.

Sample Abbreviation	Morphological Part	Month of Harvest
YS_V	Young shoots	May
FL_VI	Flowers	June
L_VI	Leaves	June
L_VII	Leaves	July
L_IX	Leaves	September
FR_V	Fruits (from last year)	May
FR_VII	Fruits (green)	July
FR_IX	Fruits (black)	September

## Data Availability

Data are contained within the article.

## Supplementary Materials

The following supporting information can be downloaded at: https://www.mdpi.com/article/10.3390/molecules29020399/s1, Table S1: The results of statistical analysis (*p* values) regarding the data of echinacoside content in *Ligustrum vulgare* extracts using one-way ANOVA with Tukey’s post-test; Table S2: The results of statistical analysis (*p* values) regarding the data of rutin (quercitin-3-*O*-rutinoside) content in *Ligustrum vulgare* extracts using one-way ANOVA with Tukey’s post-test; Table S3: The results of statistical analysis (*p* values) regarding the data of oleuropein content in *Ligustrum vulgare* extracts using one-way ANOVA with Tukey’s post-test; Table S4: The results of statistical analysis (*p* values) regarding the data of antioxidant activity by ABTS assay using one-way ANOVA with Tukey’s post-test; Table S5: The results of statistical analysis (*p* values) regarding the data of antioxidant activity by DPPH assay using one-way ANOVA with Tukey’s post-test; Table S6: The results of statistical analysis (*p* values) regarding the data of maltose content after the enzymatic reaction using one-way ANOVA with Tukey’s post-test; Table S7: The results of statistical analysis (*p* values) regarding the data of dextrin 1 content after the enzymatic reaction using one-way ANOVA with Tukey’s post-test; Table S8: The results of statistical analysis (*p* values) regarding the data of dextrin 2 content after the enzymatic reaction using one-way ANOVA with Tukey’s post-test; Table S9: The results of statistical analysis (*p* values) regarding the data of amylase inhibition using one-way ANOVA with Tukey’s post-test; Table S10: The results of statistical analysis (*p* values) regarding the data of cyclooxygenase inhibition using one-way ANOVA with Tukey’s post-test; Table S11: The EC50 values obtained from MTT assay in HT29 and HepG2 cultures after 6 h and 24 h exposure to different *Ligustrum vulgare* extracts.
