# Supplementary material for: Comparative Analysis of Phytochemical Profiles and Selected Biological Activities of Various Morphological Parts of Ligustrum vulgare
Source: Molecules. 2024 Jan 13;29(2):399. doi: 10.3390/molecules29020399 (PMC10819685; doi:10.3390/molecules29020399)
Supplement: Supplementary file 1 [file molecules-29-00399-s001.zip › molecules-2775648-supplementary.pdf]

Comparative Analysis of Phytochemical Profiles and Selected Biological Activities of Various Morphological Parts of *Ligustrum vulgare*

Szymon Litewski, Izabela Koss-Mikołajczyk and Barbara Kusznierewicz *

Table S1. The results of statistical analysis (p values) regarding the data of echinacoside content in *Ligustrum vulgare* extracts using one-way ANOVA with Tuckey post-test. The abbreviations: FL, YS, L and FR refer to the morphological part of *Ligustrum vulgare* and mean, respectively: flowers, young shoots, leaves and fruits, while the numbers IV-IX indicate the month of their harvest.

Sample	YS_V	FL_VI	L_VI	L_VII	L_IX	FR_VII	FR_IX
FL_VI	<0.0001	-					
L_VI	<0.0001	0.9989 ns	-				
L_VII	<0.0001	0.0031	0.0094	-			
L_IX	<0.0001	<0.0001	<0.0001	<0.0001	-		
FR_VII	<0.0001	<0.0001	<0.0001	<0.0001	<0.0001	-	
FR_IX	<0.0001	<0.0001	<0.0001	<0.0001	0.0004	<0.0001	-
FR_V	<0.0001	<0.0001	<0.0001	<0.0001	0.9992 ns	<0.0001	0.0011

ns – not statistically significant ($p > 0.05$).

Table S2. The results of statistical analysis (p values) regarding the data of rutin (quercitin-3-*O*-rutinoside) content in *Ligustrum vulgare* extracts using one-way ANOVA with Tuckey post-test. The abbreviations: FL, YS, L and FR refer to the morphological part of *Ligustrum vulgare* and mean, respectively: flowers, young shoots, leaves and fruits, while the numbers IV-IX indicate the month of their harvest.

Sample	YS_V	FL_VI	L_VI	L_VII	L_IX	FR_VII	FR_IX
FL_VI	0.0560 ns	-					
L_VI	0.9987 ns	0.1569 ns	-				
L_VII	0.0116	0.9892 ns	0.0356	-			
L_IX	<0.0001	<0.0001	<0.0001	0.0004	-		
FR_VII	<0.0001	<0.0001	<0.0001	<0.0001	<0.0001	-	
FR_IX	<0.0001	<0.0001	<0.0001	<0.0001	<0.0001	0.1633 ns	-
FR_V	<0.0001	<0.0001	<0.0001	<0.0001	<0.0001	0.2685 ns	0.0015

ns – not statistically significant ($p > 0.05$).

Table S3. The results of statistical analysis (p values) regarding the data of oleuropein content in *Ligustrum vulgare* extracts using one-way ANOVA with Tuckey post-test. The abbreviations: FL, YS, L and FR refer to the morphological part of *Ligustrum vulgare* and mean, respectively: flowers, young shoots, leaves and fruits, while the numbers IV-IX indicate the month of their harvest.

Sample	YS_V	FL_VI	L_VI	L_VII	L_IX	FR_VII	FR_IX
FL_VI	0.0028	-					
L_VI	0.0036	<0.0001	-				
L_VII	<0.0001	<0.0001	0.3138 ns	-			
L_IX	0.0038	>0.9999 ns	<0.0001	<0.0001	-		

FR_VII	<0.0001	<0.0001	0.0006	0.0609 ns	<0.0001	-	
FR_IX	<0.0001	<0.0001	<0.0001	<0.0001	<0.0001	0.0039	-
FR_V	<0.0001	<0.0001	<0.0001	<0.0001	<0.0001	0.0002	0.7097 ns

ns – not statistically significant (p > 0.05).

Table S4. The results of statistical analysis (p values) regarding the data of antioxidant activity by ABTS assay using one-way ANOVA with Tuckey post-test. The abbreviations: FL, YS, L and FR refer to the morphological part of *Ligustrum vulgare* and mean, respectively: flowers, young shoots, leaves and fruits, while the numbers IV-IX indicate the month of their harvest.

Sample	YS_V	FL_VI	L_VI	L_VII	L_IX	FR_VII	FR_IX
FL_VI	0.5529 ns	-					
L_VI	0.7238 ns	0.0412	-				
L_VII	>0.9999 ns	0.4442 ns	0.8231 ns	-			
L_IX	0.9992 ns	0.2765 ns	0.9478 ns	>0.9999 ns	-		
FR_VII	0.0005	<0.0001	0.0123	0.0008	0.0015	-	
FR_IX	<0.0001	0.0002	<0.0001	<0.0001	<0.0001	<0.0001	-
FR_V	0.0013	0.0518 ns	<0.0001	0.0009	0.0005	<0.0001	0.1530 ns

ns – not statistically significant (p > 0.05).

Table S5. The results of statistical analysis (p values) regarding the data of antioxidant activity by DPPH assay using one-way ANOVA with Tuckey post-test. The abbreviations: FL, YS, L and FR refer to the morphological part of *Ligustrum vulgare* and mean, respectively: flowers, young shoots, leaves and fruits, while the numbers IV-IX indicate the month of their harvest.

Sample	YS_V	FL_VI	L_VI	L_VII	L_IX	FR_VII	FR_IX
FL_VI	0.0020	-					
L_VI	0.0432	0.7530 ns	-				
L_VII	<0.0001	0.2802 ns	0.0166	-			
L_IX	0.6672 ns	0.0573 ns	0.6257 ns	0.0005	-		
FR_VII	0.0150	0.9620 ns	0.9990 ns	0.0457	0.3235 ns	-	
FR_IX	0.0003	<0.0001	<0.0001	<0.0001	<0.0001	<0.0001	-
FR_V	<0.0001	<0.0001	<0.0001	<0.0001	<0.0001	<0.0001	0.1884 ns

ns – not statistically significant (p > 0.05).

Table S6. The results of statistical analysis (p values) regarding the data of maltose content after the enzymatic reaction using one-way ANOVA with Tuckey post-test. The abbreviations: FL, YS, L and FR refer to the morphological part of *Ligustrum vulgare* and mean, respectively: flowers, young shoots, leaves and fruits, while the numbers IV-IX indicate the month of their harvest.

Sample	Control	Acarbose	YS_V	FL_VI	L_VI	L_VII	L_IX	FR_VII	FR_IX
Acarbose	<0.0001	-							
YS_V	0.0025	<0.0001	-						
FL_VI	<0.0001	<0.0001	0.0826 ns	-					
L_VI	0.0331	<0.0001	0.9645 ns	0.0067	-				
L_VII	<0.0001	<0.0001	0.3839 ns	0.9944 ns	0.0462	-			
L_IX	0.0051	<0.0001	>0.9999 ns	0.0434	0.9956 ns	0.2359 ns	-		

FR_VII	0.0021	<0.0001	>0.9999 ^{ns}	0.0945 ^{ns}	0.9502 ^{ns}	0.4220 ^{ns}	>0.9999 ^{ns}	-	
FR_IX	<0.0001	<0.0001	0.4295 ^{ns}	0.9897 ^{ns}	0.0547 ^{ns}	>0.9999 ^{ns}	0.2697 ^{ns}	0.4696 ^{ns}	-
FR_V	<0.0001	0.0015	0.0028	0.8378 ^{ns}	0.0002	0.3269 ^{ns}	0.0014	0.0033	0.2882 ^{ns}

ns – not statistically significant ($p > 0.05$).

Table S7. The results of statistical analysis (p values) regarding the data of dextrin 1 content after the enzymatic reaction using one-way ANOVA with Tuckey post-test. The abbreviations: FL, YS, L and FR refer to the morphological part of *Ligustrum vulgare* and mean, respectively: flowers, young shoots, leaves and fruits, while the numbers IV-IX indicate the month of their harvest.

Sample	Control	Acarbose	YS_V	FL_VI	L_VI	L_VII	L_IX	FR_VII	FR_IX
Acarbose	<0.0001	-							
YS_V	<0.0001	0.0088	-						
FL_VI	<0.0001	<0.0001	0.0220	-					
L_VI	<0.0001	0.1262 ^{ns}	0.9433 ^{ns}	0.0013	-				
L_VII	<0.0001	0.0573 ^{ns}	0.9952 ^{ns}	0.0032	>0.9999 ^{ns}	-			
L_IX	<0.0001	0.0004	0.9095 ^{ns}	0.3182 ^{ns}	0.2309 ^{ns}	0.4242 ^{ns}	-		
FR_VII	<0.0001	0.0011	0.9913 ^{ns}	0.1493 ^{ns}	0.4518 ^{ns}	0.6983 ^{ns}	>0.9999 ^{ns}	-	
FR_IX	<0.0001	<0.0001	0.0737 ^{ns}	0.9998 ^{ns}	0.0048	0.0116	0.6545 ^{ns}	0.3844 ^{ns}	-
FR_V	<0.0001	<0.0001	0.0029	0.9927 ^{ns}	0.0002	0.0004	0.0596 ^{ns}	0.0232	0.8648 ^{ns}

ns – not statistically significant ($p > 0.05$).

Table S8. The results of statistical analysis (p values) regarding the data of dextrin 2 content after the enzymatic reaction using one-way ANOVA with Tuckey post-test. The abbreviations: FL, YS, L and FR refer to the morphological part of *Ligustrum vulgare* and mean, respectively: flowers, young shoots, leaves and fruits, while the numbers IV-IX indicate the month of their harvest.

Sample	Control	Acarbose	YS_V	FL_VI	L_VI	L_VII	L_IX	FR_VII	FR_IX
Acarbose	<0.0001	-							
YS_V	<0.0001	0.0003	-						
FL_VI	<0.0001	0.0061	0.9291 ^{ns}	-					
L_VI	<0.0001	<0.0001	0.9454 ^{ns}	0.2637 ^{ns}	-				
L_VII	<0.0001	0.0009	>0.9999 ^{ns}	0.9956 ^{ns}	0.7503 ^{ns}	-			
L_IX	<0.0001	0.0029	0.9881 ^{ns}	>0.9999	0.4315 ^{ns}	>0.9999 ^{ns}	-		
FR_VII	<0.0001	0.0006	>0.9999 ^{ns}	0.9845 ^{ns}	0.8400 ^{ns}	>0.9999 ^{ns}	0.9991 ^{ns}	-	
FR_IX	0.0018	<0.0001	0.0005	<0.0001	0.0082	0.0002	<0.0001	0.0003	-
FR_V	0.0018	<0.0001	0.0005	<0.0001	0.0080	0.0002	<0.0001	0.0003	>0.9999 ^{ns}

ns – not statistically significant ($p > 0.05$).

Table S9. The results of statistical analysis (p values) regarding the data of amylase inhibition using one-way ANOVA with Tuckey post-test. The abbreviations: FL, YS, L and FR refer to the morphological part of *Ligustrum vulgare* and mean, respectively: flowers, young shoots, leaves and fruits, while the numbers IV-IX indicate the month of their harvest.

Sample	Control	Acarbose	YS_V	FL_VI	L_VI	L_VII	L_IX	FR_VII	FR_IX

Acarbose	<0.0001	-							
YS_V	0.0006	<0.0001	-						
FL_VI	<0.0001	<0.0001	0.0319	-					
L_VI	0.0128	<0.0001	0.9195 ^{ns}	0.0016	-				
L_VII	<0.0001	<0.0001	0.2179 ^{ns}	0.9883 ^{ns}	0.0144	-			
L_IX	0.0014	<0.0001	>0.9999 ns	0.0143	0.9886 ^{ns}	0.1116 ^{ns}	-		
FR_VII	0.0029	<0.0001	0.9992 ^{ns}	0.0072	0.9993 ^{ns}	0.0601 ^{ns}	>0.9999 ns	-	
FR_IX	<0.0001	<0.0001	0.2739 ^{ns}	0.9724 ^{ns}	0.0195	>0.9999 ns	0.1448 ^{ns}	0.0795 ^{ns}	-
FR_V	<0.0001	0.0002	0.0006	0.7067 ^{ns}	<0.0001	0.1870 ^{ns}	0.0003	0.0001	0.1457 ^{ns}

ns – not statistically significant (p > 0.05).

Table S10. The results of statistical analysis (p values) regarding the data of cyclooxygenase COX-2 inhibition using one-way ANOVA with Tuckey post-test. The abbreviations: FL, YS, L and FR refer to the morphological part of *Ligustrum vulgare* and mean, respectively: flowers, young shoots, leaves and fruits, while the numbers IV-IX indicate the month of their harvest.

Sample	Control	Inhibitor	YS_V	FL_VI	L_VI	L_VII	L_IX	FR_VII	FR_IX
Inhibitor	<0.0001	-							
YS_V	<0.0001	0.0812 ^{ns}	-						
FL_VI	<0.0001	0.5003 ^{ns}	0.9765 ^{ns}	-					
L_VI	<0.0001	<0.0001	0.0142	0.0012	-				
L_VII	<0.0001	0.2983 ^{ns}	0.9988 ^{ns}	>0.9999 ns	0.0028	-			
L_IX	<0.0001	0.9972 ^{ns}	0.3375 ^{ns}	0.9275 ^{ns}	<0.0001	0.7653 ^{ns}	-		
FR_VII	<0.0001	0.1643 ^{ns}	>0.9999 ns	0.9988 ^{ns}	0.0062	>0.9999 ns	0.5502 ^{ns}	-	
FR_IX	<0.0001	<0.0001	0.0891 ^{ns}	0.0087	0.9950 ^{ns}	0.0196	0.0005	0.0419	-
FR_V	<0.0001	<0.0001	<0.0001	<0.0001	0.0015	<0.0001	<0.0001	<0.0001	0.0002

ns – not statistically significant (p > 0.05).

Table S11. The EC50 values obtained from MTT assay in HT29 and HepG2 cultures after 6 h and 24 h exposure to different *Ligustrum vulgare* extracts. The abbreviations: FL, YS, L and FR refer to the morphological part of *Ligustrum vulgare* and mean, respectively: flowers, young shoots, leaves and fruits, while the numbers IV-IX indicate the month of their harvest.

Sample	EC50 [mg d.w./mL]			
	HT29		HepG2	
	6h	24h	6h	24h
YS_V	2,0	1,2	2,0	0,8
FL_VI	-	3,5	1,5	1,0
L_VI	1,9	1,8	1,0	0,8
L_VII	1,8	1,0	1,8	0,8
L_IX	3,5	2,0	4,0	1,5
FR_VII	2,5	1,5	1,5	0,5
FR_IX	3,8	3,0	3,6	1,0

FR_V	-	-	3,2	1,2
